# The normal breast microenvironment of premenopausal women differentially influences the behavior of breast cancer cells *in vitro *and *in vivo*

**DOI:** 10.1186/1741-7015-8-27

**Published:** 2010-05-21

**Authors:** Jodie M Fleming, Tyler C Miller, Mariam Quinones, Zhen Xiao, Xia Xu, Matthew J Meyer, Erika Ginsburg, Timothy D Veenstra, Barbara K Vonderhaar

**Affiliations:** 1 Mammary Biology and Tumorigenesis Laboratory, Center for Cancer Research, National Cancer Institute, Bethesda, MD, USA; 2 Bioinformatics and Computational Biosciences Branch, The Office of Science Management and Operations, National Institute of Allergy and Infectious Disease, Bethesda, MD, USA; 3 Laboratory of Proteomics and Analytical Technologies, Advanced Technology Program, SAIC-Frederick, NCI-Frederick, Frederick, MD, USA

## Abstract

**Background:**

Breast cancer studies frequently focus on the role of the tumor microenvironment in the promotion of cancer; however, the influence of the normal breast microenvironment on cancer cells remains relatively unknown. To investigate the role of the normal breast microenvironment on breast cancer cell tumorigenicity, we examined whether extracellular matrix molecules (ECM) derived from premenopausal African-American (AA) or Caucasian-American (CAU) breast tissue would affect the tumorigenicity of cancer cells *in vitro *and *in vivo*. We chose these two populations because of the well documented predisposition of AA women to develop aggressive, highly metastatic breast cancer compared to CAU women.

**Methods:**

The effects of primary breast fibroblasts on tumorigenicity were analyzed via real-time PCR arrays and mouse xenograft models. Whole breast ECM was isolated, analyzed via zymography, and its effects on breast cancer cell aggressiveness were tested *in vitro *via soft agar and invasion assays, and *in vivo *via xenograft models. Breast ECM and hormone metabolites were analyzed via mass spectrometry.

**Results:**

Mouse mammary glands humanized with premenopausal CAU fibroblasts and injected with primary breast cancer cells developed significantly larger tumors compared to AA humanized glands. Examination of 164 ECM molecules and cytokines from CAU-derived fibroblasts demonstrated a differentially regulated set of ECM proteins and increased cytokine expression. Whole breast ECM was isolated; invasion and soft agar assays demonstrated that estrogen receptor (ER)^-^, progesterone receptor (PR)/PR^- ^cells were significantly more aggressive when in contact with AA ECM, as were ER^+^/PR^+ ^cells with CAU ECM. Using zymography, protease activity was comparatively upregulated in CAU ECM. In xenograft models, CAU ECM significantly increased the tumorigenicity of ER^+^/PR^+ ^cells and enhanced metastases. Mass spectrometry analysis of ECM proteins showed that only 1,759 of approximately 8,000 identified were in common. In the AA dataset, proteins associated with breast cancer were primarily related to tumorigenesis/neoplasia, while CAU unique proteins were involved with growth/metastasis. Using a novel mass spectrometry method, 17 biologically active hormones were measured; estradiol, estriol and 2-methoxyestrone were significantly higher in CAU breast tissue.

**Conclusions:**

This study details normal premenopausal breast tissue composition, delineates potential mechanisms for breast cancer development, and provides data for further investigation into the role of the microenvironment in cancer disparities.

## Background

Breast cancer is the most frequently diagnosed cancer and the second leading cause of cancer-related deaths of women living in the US [1]. Breast cancer manifests itself in the mammary epithelium; however, tumors do not progress to malignancy in isolation. The local microenvironment can enhance or suppress tumor growth and progression, as well as metastases [2-8]. The stroma is composed of diverse cell types including endothelial and immune cells, adipocytes, and fibroblasts. These cells secrete products, including growth factors and extracellular matrix (ECM) components, which profoundly affect cell behavior. It has been suggested that altered communication between these cells can lead to the progression or expansion of malignant growth. While numerous studies have observed the effects of synthetic or mouse-derived ECM on breast cancer cells, relatively little is known about the molecular interactions between human breast ECM and epithelial cells.

Recently, a novel *in vivo *xenograft model mimicking human ductal carcinoma *in situ *(DCIS) illustrated that the progression of normal tissue towards a neoplastic state depends on the surrounding stromal cells [9]. Normal myoepithelial cells inhibited the progression of DCIS to an invasive carcinoma, while the addition of fibroblasts enhanced invasion. Additionally, reports demonstrated that the mammary microenvironment can reprogram both embryonic and adult murine stem cells into mammary cells capable of expressing milk proteins and hormone receptors, substantiating the remarkable control the environment has on cell behavior [10,11].

African-American (AA) women have a lower overall incidence of breast cancer compared to Caucasian-American (CAU) women but a significantly higher incidence rate before the age of 40, and a higher mortality rate at any age [1]. Breast carcinomas in premenopausal AA patients are more often triple negative, which lack estrogen receptor (ER), progesterone receptor (PR), and human epidermal growth factor receptor 2 (HER2) amplification [12-15]. Triple-negative cancers constitute one of the most challenging types of breast cancer, as the only systemic therapy is chemotherapy.

It has been proposed that premenopausal AA women develop triple-negative tumors due to multifactorial differences including socioeconomic factors, body mass index, diet, earlier age at first pregnancy, lower incidence of breastfeeding, and higher breast density [16,17]. However, these factors do not explain everything. A recent study reported that even after adjusting for socioeconomic status, AA women still have a 22% higher mortality rate [18]. Interestingly, there are parallels in carcinoma development between women in western African nations and AAs, including early onset of disease and hormone receptor negativity [19]. These women share common ancestry suggesting that mutations in breast cancer susceptibility genes are partly responsible for the high prevalence of triple-negative carcinomas [19]. This predisposition of AA women to develop a more aggressive cancer compared to CAU women provides a unique model for studying the role of the normal breast microenvironment on breast cancer development. Hence, our objective was to determine whether factors within the local microenvironment of premenopausal AA and CAU women differentially alter the behavior of breast cancer cells.

In this study, premenopausal AA or CAU primary breast fibroblasts and ECM from whole breast tissue were isolated and examined by several *in vitro *and *in vivo *methods. ER^-^/PR^- ^cells were significantly more aggressive in the presence of AA ECM by both invasion and soft agar assays; in contrast, CAU ECM caused increased aggressiveness with ER^+^/PR^+ ^cells. By mass spectrometry, approximately 22% of identified proteins were common to both AA-derived and CAU-derived ECM; proteins related to tumorigenesis/neoplasia were more highly associated with the AA ECM while proteins involved with growth/metastasis were more prevalent with the CAU ECM. Using a novel mass spectrometry assay to measure biologically active hormones, only estradiol, estriol, and 2-methoxyestrone levels were significantly higher in the CAU breast. Finally, in a xenograft model, CAU ECM significantly enhanced the tumorigenicity and metastases of ER^+^/PR^+ ^cells. To our knowledge, we are the first to investigate the normal ECM of premenopausal women; furthermore, results from this study may help identify mechanisms by which AA are predisposed to developing a more aggressive breast cancer.

## Methods

### Collection and processing of patient samples

Collection of patient samples was performed in accordance with the guidelines of the National Cancer Institute Review Board, under four separate approved protocol numbers OH99-C-NO57, 02-C-0077E, 04-C-0199, and OHSR4789. Written informed consent was obtained from all human subjects as stipulated in the protocols. Breast tissue was collected from fasting, age-matched, premenopausal AA or CAU reduction mammoplasty patients. The tissue obtained for analyses was considered pathological medical waste; thus any clinical details of the women, apart from age and race, were unattainable. Overall, 53 AA and 50 CAU breast tissue samples, from patients with a median age of 29 years, were used for analyses. Race was self-reported by the patients. Tissue was collected from southern, eastern, and midwestern regions of the US. A pathologist confirmed that each patient was free of malignant or hyperplasic growth. Immediately after surgery a separate piece of tissue was used for isolation of primary human breast fibroblasts, and the remaining tissue was snap frozen and stored at -80°C for RNA and protein analyses, and for ECM isolation.

Pleural effusion cells were collected from a parous, 49-year-old Caucasian breast cancer patient with an ER^+^/PR^+^, Her2^-^, T1, pN1, M1, Grade 3, poorly differentiated invasive ductal carcinoma. Immediately following collection, cells were processed as follows: cells were gently pelleted by centrifugation, washed twice in Hank's buffered saline solution, frozen viably in dimethylsulfoxide (DMSO) Freeze media (Invitrogen; Gaithersburg, MD, USA) and stored in liquid nitrogen until used. The cells derived from the pleural effusion were ER^-^/PR^- ^and Her2^-^, as determined by immunohistochemistry.

### Fluorescent activated cytometric sorting (FACS)

Immediately prior to use, pleural effusion cells were stained with lineage markers to segregate tumor from non-tumor cells as previously described [20]. Briefly, lineage marker antibodies used were fluorescein conjugated anti-human CD2, CD3, CD10, CD16, CD18, CD31, CD64, and CD140b (BD Biosciences, San Jose, CA, USA). Cells were stained in a phosphate-buffered saline (PBS) solution containing 0.1% fetal bovine serum (FBS) and 100 units/ml penicillin/streptomycin for 25 min at 4°C. Cell sorting was performed on a BD FACSAria operating at low pressure (20 psi) using a 100 μm nozzle. Doublets were electronically gated out and 7-aminoactinomycin D (7AAD, 1 μg/ml final concentration, BD Biosciences) was used for live/dead cell distinction. Live, fluorescein negative tumor cells were sorted into a PBS solution containing 50% FBS. Post-sort analysis typically indicated purities of >96% with minimal cell death (<10%). FACS data were analyzed using FlowJo v8.7.3 (TreeStar, Ashland, OR, USA).

### Cell culture

MCF10Ca1h cells (kind gift of FR Miller, Wayne State University, Detroit, MI, USA, through LM Wakefield, Center for Cancer Research (CCR), National Cancer Institute (NCI), Bethesda, MD, USA) were maintained as described previously [21]. All other cell lines were obtained from the American Type Culture Collection (ATCC; http://www.atcc.org) and cultured according to the repository's instructions. Fibroblasts were isolated as described [22]. Briefly, <5 mm pieces of tissue were placed on a scratched cell culture dish. Tissue pieces were covered with a minimal amount of media and, with time, the fibroblasts crawled out of the tissue to form a monolayer on the dish. The fragments of tissue were removed and the remaining fibroblasts were passaged and plated as monolayer cultures to expand and ensure fibroblast purity. When necessary, epithelial cells were separated from the stromal cells by differential trypsinization and selective pressure with fibroblast growth medium. Fibroblasts were grown for a maximum of two passages prior to analysis.

### Isolation of whole breast tissue ECM proteins

Extraction of human breast ECM from whole breast tissue was performed as previously described [23]. A minimum of three different age-matched patient samples per treatment group was used for each extraction (total n = 26 AA, and 21 CAU). Pools were necessary in order to obtain enough tissue from which to extract ECM. A different pool of samples was used for each experiment. Matrices were stored on ice at 4°C and used within 5 days of isolation.

### Zymography

Equal amounts of protein were separated by gel electrophoresis in a 10% Tris-glycine polyacrylamide gel (Invitrogen) with 0.1% gelatin incorporated as a substrate. Proteins were renatured, soaked in developing buffer, and then stained according to the manufacturer's instructions. Matrix metalloproteinase (MMP) activity was visualized as clear bands against a dark blue background where the protease has digested the substrate. Identification of MMPs was based on published molecular weights. Three independent experiments, each with different pools of age-matched patient samples (minimum of three patient samples per pool), were performed with each individual experiment repeated in duplicate to ensure repeatability.

### Invasion assay

Transwell membranes (8 μm pores) were precoated with equal amounts of ECM, adjusted for total protein content. Breast cancer cells were washed, resuspended in serum-free medium, and then plated in the top chamber of transwell inserts (at the predetermined concentration for each cell line). The cells were allowed to invade through the membrane for up to 16 h towards FBS-containing medium in the bottom chamber. Following invasion, the cells were wiped from the top surface of the membrane; the remaining cells were fixed in methanol and stained with a 1% toluidine blue solution. Four independent experiments, each with different pools of patient samples (minimum of three patient samples per pool), were performed with each individual experiment repeated in duplicate to ensure repeatability.

### Soft agar assay

Breast cancer cells were plated on an 0.66% agarose base in a 0.33% top soft agar layer in 35 mm cell culture dishes with the addition or absence of 100 μl of ECM, adjusted for equal protein content. Cells were incubated for 10 to 12 days, and then stained overnight with nitrobluetetrazolium. The total number of colonies in each dish was counted using the AccuCount 1000 colony counter (BioLogics, Manassas, VA, USA); however, only colonies over 1 μm in diameter were included in the calculation. Three independent experiments, each with different pools of patient samples (minimum of three patient samples per pool), were performed with each individual experiment repeated in duplicate to ensure repeatability.

### Immunohistochemistry

Immunohistochemistry was performed with appropriate controls as described previously [24]. Briefly, sections of formalin fixed, paraffin embedded tissue 5 μm thick were prepared from all tumors obtained in the xenograft studies, and fragments of the lungs and livers of animals used in the metastasis experiments. The human specific COXIV antibody (1:1,000, Cell Signaling; Boston, MA, USA) was used for detection of metastases of breast cancer cells in the xenograft experiments. Antibodies Ki67 (Santa Cruz Biotechnology, Santa Cruz, CA, USA) and ER (Leica Microsystems, Bannockburn, IL, USA) were used according to manufacturers' instructions. Staining was performed using Vectastain ABC kit (Vector Laboratories; Burlingame, CA, USA) according to the manufacturer's instructions. Color was developed with diaminobenzidine peroxidase substrate kit (Vector Laboratories) and sections were counterstained with hematoxylin.

### Quantitative real-time (qRT) PCR and PCR arrays

Total RNA was isolated from primary breast fibroblasts using the Qiagen RNeasy kit according to the manufacturer's instructions (Valencia, CA, USA). RNA was reverse transcribed using MMLV reverse transcriptase (Invitrogen) and primed with oligo-dT and random hexamers (Invitrogen). The cDNA was subjected to RT-PCR amplification using gene specific primers and 2 × Brilliant II Sybr Green QPCR Mastermix (Stratagene, La Jolla, CA, USA). Primer sequences are given in Additional file 1: Table S1. Quantitative RT-PCR was analyzed via the ΔΔCT method, and PCR products were visualized by agarose gel electrophoresis. qRT-PCR arrays were performed and analyzed with the commercially available qRT-PCR array kits according to the manufacturer's instructions (SABiosciences, Frederick, MD, USA). Three pools of fibroblasts, each with a minimum of three different patient fibroblasts per pool, were used for each array (n = 9 AA and 10 CAU). Validation of the array data used different, freshly isolated individual primary fibroblasts (n = 9 AA and 9 CAU).

### *In vivo *tumor formation assays

Animal experiments were conducted in accord with accepted standards of humane animal care and approved by the Animal Care and Use Committee at the National Institutes of Health, USA. Female, 8-week-old athymic Nu/Nu mice, or NOD/SCID where indicated, were randomized into three groups with a minimum of five mice per group (APA, Frederick, MD, USA). Mice were anesthetized by an intraperitoneal injection of ketamine/xylazine (750 and 50 mg/kg body weight, respectively) in Hank's buffered saline solution (HBSS) prior to surgically exposing the gland for injection. NOD SCID mice were supplemented with estrogen via a subcutaneous pellet (0.72 mg β-estradiol, 90-day release, Innovative Research of America, Sarasota, FL, USA) at the time of breast cancer cell injection. For fibroblast studies, mouse abdominal mammary glands were humanized with primary human fibroblasts as previously described [25]. Each experiment used a minimum of three different patient pools of fibroblasts per humanization (total AA n = 12, CAU n = 14). Following humanization, primary metastatic breast cancer cells, derived from a pleural effusion, were sorted via FACS to remove non-epithelial cells, and then mixed with 1:1 ratio of 1 × PBS:Matrigel (BD Biosciences). A total of 30 μl of ECM containing 5 × 10^3 ^cells was injected into the humanized abdominal mammary gland fat pad. Tumor growth was measured using calipers on a weekly basis. Tumors were excised when the majority of tumors reach 1.0 cm^3^, and final tumor volume was calculated ((0.5 × L) × (0.5 × W) × (0.5 × H) × (4/3) × (π)).

For ECM studies, breast cancer cells (MDA-MB-231 and T47D) proliferating in log phase were mixed with control matrix (Matrigel), AA or CAU ECM, adjusted for equal protein content. A total of 40 μl of ECM containing 1 × 10^6 ^or 2 × 10^6 ^cells was injected, respectively, into the abdominal mammary fat pad or subcutaneously proximal to the scapula. Tumor growth was measured on a weekly basis using calipers. Tumors were excised using survival surgery when the majority of tumors reach 1.0 cm^3^, and final tumor volume was calculated. At 3 months post tumor excision, the animals were killed and the liver and lung tissues were removed for detection of metastases. Tissues were analyzed for metastases by pathological evaluation, quantitative PCR using human-specific primers developed to β2-microglobulin [26], and immunohistochemistry using a human specific COXIV antibody. Each animal experiment was repeated a minimum of two times, using different pools of ECM (minimum of three patients per pool) for each experiment.

### Mass spectrometry

Three sets of pools of AA and CAU ECM, derived from different patients in each pool, minimum of three patients per pool, were quantified and 2 μg of ECM from each pool were separated on a 4% to 12% Nu-PAGE Bis-Tris gel in MOPS SDS running buffer (Invitrogen). The gel was washed and stained using SimplyBlue Safe Stain Solution (Invitrogen). Each gel lane was divided into 10 sections, excised, destained, lyophilized and digested with trypsin in 25 mM NH_4_HCO_3_, pH 8.4, overnight at 37°C. The tryptic peptides were extracted from gel slices using 70% acetonitrile containing 5% formic acid, lyophilized, and the peptides reconstituted in 0.1% formic acid prior to nanoflow reversed-phase liquid chromatography (nanoRPLC) mass spectrometry analysis. NanoRPLC columns were slurry packed with 5 μm, 300 Å pore size C-18 silica-bonded stationary reverse-phase particles (Jupiter; Phenomenex, Torrance, CA, USA) in a 75 μm internal diameter × 10 cm fused silica capillary with a flame pulled tip. The column was connected to an Agilent 1100 nanoLC system and coupled to a linear ion trap (LIT) mass spectrometer (LTQ, ThermoElectron, , San Jose, CA, USA, operated with Xcalibur 1.4 SR1 software). The samples were injected onto the column and the peptides eluted using a gradient of mobile phase A (0.1% formic acid in water) and B (0.1% formic acid in acetonitrile). The LTQ was operated in a data-dependent mode in which the seven most abundant peptide molecular ions in every MS scan were sequentially selected for collision-induced dissociation (CID) using a normalized collision energy of 35%. Dynamic exclusion was applied to minimize repeated selection of peptides previously selected for CID.

Tandem mass spectra were searched against the UniProt human proteomic database from the European Bioinformatics Institute with SEQUEST (http://fields.scripps.edu/sequest/) operating on a 40-node Beowulf cluster. Peptides were searched using fully tryptic cleavage constraints. Oxidation of methionine (+15.9949 Da) was included as dynamic modification. For a peptide to be considered legitimately identified, it must have achieved a minimum Δ correlation (ΔC_n_) of 0.08 and charge state-dependent cross correlation (Xcorr) scores of 1.9 for [M + H]^1+^, 2.2 for [M + 2H]^2+^, and 3.1 for [M + 3H]^3+ ^peptide molecular ions. Data were subjected to functional analysis through the use of Ingenuity pathways analysis (IPA; Ingenuity Systems, http://www.ingenuity.com) and BIOBASE (http://www.biobase-international.com).

### Estrogen metabolite analysis

#### Reagents and materials for steroid analysis

A total of 15 estrogens including estrone (E_1_), estradiol (E_2_), estriol (E_3_), 16-epiestriol (16-epiE_3_), 17-epiestriol (17-epiE_3_), 16-ketoestradiol (16-ketoE_2_), 16α-hydroxyestrone (16α-OHE_1_), 2-methoxyestrone (2-MeOE_1_), 4-methoxyestrone (4-MeOE_1_), 2-hydroxyestrone-3-methyl ether (3-MeOE_1_), 2-methoxyestradiol (2-MeOE_2_), 4-methoxyestradiol (4-MeOE_2_), 2-hydroxyestrone (2-OHE_1_), 4-hydroxyestrone (4-OHE_1_), and 2-hydroxyestradiol (2-OHE_2_) and 2 androgens, androstenedione and testosterone, were obtained from Steraloids (Newport, RI, USA). Stable isotope labeled steroids, including estradiol-13,14,15,16,17,18-^13^C_6 _(^13^C_6_-E_2_) and estrone-13,14,15,16,17,18-^13^C_6 _(^13^C_6_-E_1_) were purchased from Cambridge Isotope Laboratories (Andover, MA, USA); estriol-2,4,17-*d*_3 _(d_3_-E_3_), 2-hydroxyestradiol-1,4,16,16,17-*d*_5 _(d_5_-2-OHE_2_), 2-methoxyestradiol-1,4,16,16,17-*d*_5 _(d_5_-2-MeOE_2_), androstenedione-2,2,4,6,6,16,16-*d*_7 _and testosterone-16,16,17-*d*_3 _were obtained from C/D/N Isotopes (Pointe-Claire, Quebec, Canada). 16-Epiestriol-2,4,16-*d*_3 _(d_3_-16-epiE_3_) was purchased from Medical Isotopes (Pelham, NH, USA). All steroid analytical standards have reported chemical and isotopic purity ≥98%, and were used without further purification. Dichloromethane and methanol were obtained from EM Science (Gibbstown, NJ, USA). Glacial acetic acid and sodium bicarbonate were purchased from JT Baker (Phillipsburg, NJ, USA) and sodium hydroxide and sodium acetate were purchased from Fisher Scientific (Fair Lawn, NJ, USA). Ethyl alcohol was obtained from Pharmco Products (Brookfield, CT, USA). Formic acid, acetone, dansyl chloride, and L-ascorbic acid were obtained from Sigma-Aldrich (St Louis, MO, USA). All chemicals and solvents used in this study were high performance liquid chromatography (HPLC) or reagent grade unless otherwise noted.

#### Preparation of stock and working standard solutions

Stock solutions of steroids and stable isotope labeled steroids were each prepared at 80 μg/ml by dissolving 2 mg of each estrogen powder in methanol containing 0.1% l-ascorbic acid to a final volume of 25 ml in a volumetric flask. Stock solutions were monitored by measuring the absolute peak height of each steroid using liquid chromatography-mass spectrometry/mass spectrometry (LC-MS/MS) to verify that no time-dependent degradation of steroid standards had occurred. The stock solutions are stable for at least 2 months while stored at -20°C. Working standard solutions of steroids at 0.32 and 8.0 ng/ml were prepared by dilutions of the stock solutions with methanol containing 0.1% l-ascorbic acid.

#### Sample preparation procedure

To quantitatively measure unconjugated biologically active estrogen metabolites (EM) and androgens, breast tissue samples (0.2-0.3 g per patient) were thawed briefly at room temperature, minced with scissors, and transferred into 1.5 ml Eppendorf tubes. A total of 19 AA patient samples and 20 CAU samples were analyzed. The tissue was hardened by snap freezing in liquid nitrogen for 5 min, pulverized and then transferred into a clean screw-capped glass tube containing 1 ml of ice-cold 12.5 mM NH_4_HCO_3 _buffer. The tissue was homogenized on ice using a Tissue Tearor (Cole-Parmer, Vernon Hills, IL, USA) at low and high speed in two consecutive 15 s segments for a total of 30 s, and further sonicated on ice for five cycles of 10 s pulses with 10 s breaks in between pulses. Then, 8 ml of ethanol:acetone and 50 μl each of stable isotope-labeled estrogen and androgen internal standards (0.32 ng/ml working standard solutions) were added to each tissue homogenate. The mixture was incubated on a rotator at room temperature for 1 h and centrifuged at 3,000 *g *for 30 min. The ethanol:acetone tissue extract was transferred to a clean glass tube and dried under nitrogen gas at 60°C for 1 h (Reacti-Vap III, Pierce, Rockford, IL, USA). The residue was redissolved in 4 ml of methanol, vortexed for 1 min, chilled at -80°C for 1 h, returned to room temperature and then centrifuged at 3,000 *g *for 20 min. The methanolic phase was transferred to a clean glass tube and dried under nitrogen gas. The residue was further redissolved in 100 μl of ethanol and vortexed briefly. This was followed by the addition of 1.5 ml of 100 mM sodium acetate buffer, pH 4.6 and 5 ml of dichloromethane to the residue, and incubation at room temperature on a rotator for 30 min. The extract was chilled at -80°C for 10 min, returned to room temperature and centrifuged at 3,000 *g *for 20 min. The dichloromethane phase was transferred to a clean tube and dried. To each dried sample, 32 μl of 0.1 M sodium bicarbonate buffer, pH 9.0, and 32 μl of dansyl chloride solution (1 mg/ml in acetone) were added. After vortexing for 10 s, samples were heated at 70°C (Reacti-Therm III Heating Module; Pierce) for 10 min to form the EM and d-EM dansyl derivatives. The dansyl derivatization method modifies the phenol hydroxyl group of EM and will not react with testosterone. After derivatization, all samples were centrifuged at 3,000 *g *for 20 min, and analyzed by the capillary LC-ESI-MS/MS.

#### Capillary liquid chromatography-electrospray ionization tandem mass spectrometry analysis (Cap LC-ESI-MS/MS)

Capillary LC-ESI-MS/MS analysis was performed using an Agilent 1200 series nanoflow LC system (Agilent Technologies, Palo Alto, CA, USA) coupled to a TSQ Quantum Ultra triple quadrupole mass spectrometer (ThermoElectron). The LC separation was carried out on a 150 mm long × 300 μm internal diameter column packed with 4 μm Synergi Hydro-RP particles (Phenomenex) and maintained at 40°C. A total of 8.0 μl of each sample was injected onto the column. The mobile phase, operating at a flow rate of 4.0 μl/min, consisted of methanol as solvent A and 0.1% (v/v) formic acid in water as solvent B. A linear gradient increasing from 72% to 85% solvent A in 75 min was employed for the separation. The MS conditions were source: ESI; ion polarity: positive; spray voltage: 3,500 V; sheath and auxiliary gas: nitrogen; sheath gas pressure: 7 arbitrary units; ion transfer capillary temperature, 270°C; scan type: selected reaction monitoring (SRM); collision gas: argon; collision gas pressure: 1.5 mTorr; scan width: 0.7 u; scan time: 0.50 s; Q1 peak width: 0.70 u full-width half-maximum (FWHM); Q3 peak width: 0.70 u FWHM. The specific SRM transitions of protonated androgens were: testosterone *m/z *289→97 and 109; testosterone-*d*_3 _*m/z *292→97 and 109; androstenedione *m/z *287→97 and 109; androstenedione-*d*_7 _*m/z *287→100 and 113.

### Quantitation of tissue estrogens and androgens

Quantitation of tissue estrogens and androgens was carried out using Xcalibur Quan Browser (ThermoElectron). Briefly, calibration curves for each steroid were constructed by plotting non-labeled steroid/stable isotope labeled steroid peak area ratios obtained from calibration standards versus amounts of the steroid injected on the column and fitting these data using linear regression with 1/X weighting. The amounts of steroid in the tissue were then interpolated using this linear function.

### Statistical analysis

Pools of patient samples were necessary to obtain the required amount of tissue for ECM extraction. When appropriate, data was evaluated for significance via two-tailed Student t tests, repeated measures analysis of variance (ANOVA) with the Bonferroni multiple comparisons *post hoc *analysis, Wilcoxon matched pairs, or Mann-Whitney tests using GraphPad InStat Software version 3.0b (San Diego, CA, USA). Data was considered significant at *P *< 0.05.

## Results

### Effects of premenopausal breast fibroblasts on breast cancer cell tumorigenicity

**Figure 1:**
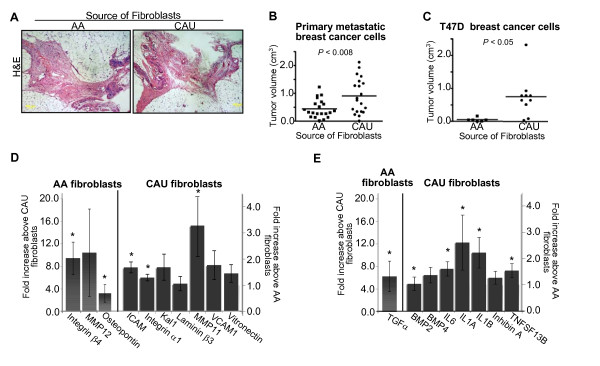
**Tumor formation and extracellular matrix molecules (ECM)/cytokine analyses of premenopausal African-American and Caucasian-American breast fibroblasts**. **(a) **Hematoxylin and eosin (H&E) staining of murine mammary glands humanized with African-American (AA) or Caucasian-American (CAU) fibroblasts. **(b,c) **Humanized murine mammary glands were injected with either primary metastatic breast cancer or T47D cells. Data represent mean ± SD of final tumor volume. **(d,e) **Quantitative real-time PCR analysis: primary human breast fibroblasts were isolated from fresh reduction mammoplasty breast tissue. RNA was isolated and analyzed via quantitative real-time PCR. Individual patient samples (*n *= 18) were used to validate array results with newly designed primers to the indicated genes. Data represent fold increase ± standard error of differentially regulated genes. *P < 0.05

It has previously been shown that tumor-derived fibroblasts promote, while normal fibroblasts inhibit, the growth of tumorigenic epithelial cells *in vitro *and *in vivo *[7,27]. Therefore, we first 'humanized' the mouse mammary gland [27] creating a microenvironment with supportive stromal components from either the AA or CAU breast. Primary breast fibroblasts from age-matched, premenopausal women were isolated [22] and a pool of a minimum of three patient-derived fibroblasts per group was injected into mouse abdominal mammary glands. A different pool of patient fibroblasts was used for each of three independent humanization experiments. As shown in Figure 1a, both the AA and CAU fibroblasts equally humanized the glands, suggesting that the source of the fibroblasts did not have an effect on the percentage of growth throughout the glands.

Following humanization, primary metastatic breast cancer cells (ER^-^/PR^-^, as described in Methods) were injected into the glands. Injection of breast cancer cells into the humanized glands was repeated in two independent experiments using different pools of patient-derived normal breast fibroblasts; however, the same breast cancer patient sample was used specifically to minimize any confounding effects of the relatively uncharacterized primary breast cancer cells. For all treatments tested, the tumor incidence was 100%, with no obvious morphological differences between treatment groups; tumors were ER^-^/PR^-^, undifferentiated invasive carcinomas. Interestingly, glands humanized with CAU fibroblasts repeatedly developed significantly larger tumors compared to AA humanized glands (Figure 1b, *P *< 0.008) suggesting that either CAU fibroblasts were more permissive to tumorigenesis, or that AA fibroblasts were more restrictive. There was no significant difference in the Ki67 proliferation index at the time of tumor collection (Additional file 1, Figure S1a). Additionally, of the tumors that contained necrotic regions, there was no significant difference in the percentage of necrotic area; however, it should be noted that the significantly larger tumors from glands humanized with CAU fibroblasts had overall higher levels of tumors containing necrotic regions compared to the AA humanized glands (50% vs 33%, Additional file 1, Figure S1b).

A similar pattern of tumor formation was observed using T47D, a well characterized, ER^+^/PR^+ ^human breast cancer cell line; the cells injected into the glands humanized with the CAU fibroblasts had increased tumor volume (Figure 1c, *P *< 0.05). The resultant tumors from both treatment groups were ER^+^/PR^+ ^by immunohistochemistry (data not shown); therefore the hormonal status of the cells was retained regardless of the source of fibroblasts.

In the presence of fibroblasts derived from the two groups, differences in tumorigenicity were observed. Therefore, the expression profiles of 164 ECM molecules and cytokines were examined using qRT-PCR arrays. Cytokines were analyzed since components of the immune system have been reported to modulate the initiation and progression of breast cancer, including metastasis to bone [28-32]. Fibroblasts were expanded in culture for less than two passages prior to isolation of total RNA. Different pools of at least three patient samples per group were used in each of three independent array experiments (n = 9 AA, n = 10 CAU). A set of ECM proteins and cytokines was consistently differentially regulated between the two groups (Additional file 1, Figure S2a and b, respectively). Array results were validated by qRT-PCR; expression patterns were observed not only using samples from the array analysis, but also with additional randomly chosen, freshly isolated, individual patient samples (n = 18, Figure 1d, e). As expected, there was substantial individual patient variation; however, 13 of the 18 genes that were differentially expressed between the groups in the array analysis were significantly different when individual patient samples were analyzed (*P *< 0.05). Of note, CAU-derived fibroblasts repeatedly exhibited an increase in cytokine expression (Figure 1e and Additional file 1, Figure S2b). This observation may explain, in part, the increase in tumor growth observed in the xenograft experiments (Figure 1b, c). Collectively, these data suggest that when stromal fibroblasts are confronted with the invasion of tumorigenic breast cells, genetic variation between the groups differentially affected tumorigenesis via the production of distinctive ECM molecules and cytokines.

### Effects of premenopausal breast ECM on breast cancer cells *in vitro*

**Figure 2:**
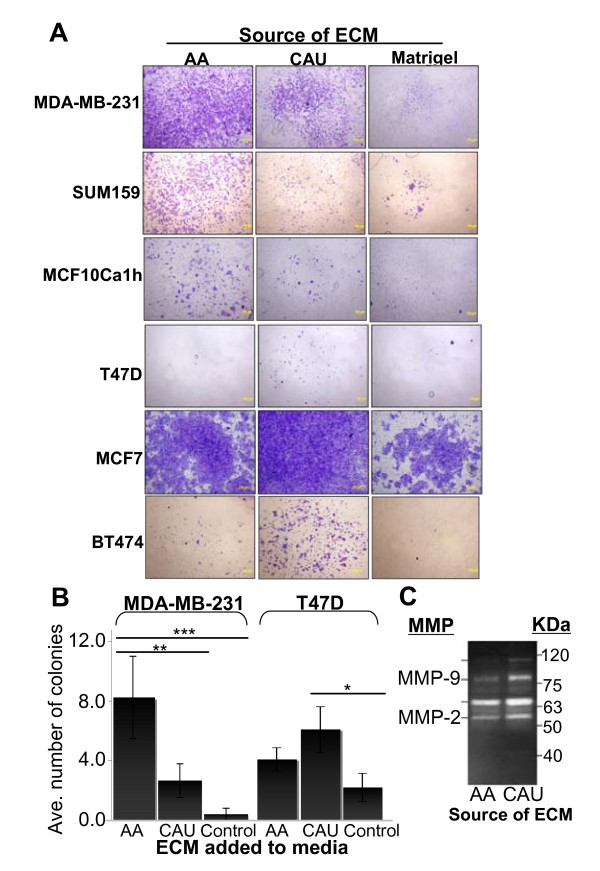
**Invasion and anchorage-independent growth of breast cancer cells in the presence of premenopausal African-American or Caucasian-American breast extracellular matrix molecules (ECM)**. **(a) **Cell invasion through transwell inserts, precoated with equal amounts of ECM. Data are representative of four independent experiments. **(b) **Soft agar assays. Cells were stained with nitrobluetetrazolium before counting after 10 days of growth. The entire dish was analyzed and colonies larger than 1 μm in diameter were counted. Data represent mean ± standard error of three separate experiments. *P < 0.05 **P < 0.01, ***P < 0.001; paired t test. **(c) **Gelatin zymography. ECM was isolated from six pools of 3-7 patients/pool. Results are representative of three experiments performed in triplicate. AA = African-American; CAU = Caucasian-American.

Numerous cell types, in addition to fibroblasts, comprise the breast microenvironment. To gain further insight into these multicellular interactions, we analyzed a broader spectrum of molecules within the breast by isolating ECM proteins from premenopausal whole breast tissue. In order to test whether ECM isolated from whole breast tissue was able to differentially influence breast cancer cell aggressiveness, we examined cell motility and invasiveness using transwell filter assays. Two classes of breast cancer cell lines were used: ER^-^/PR^- ^(MDA-MB-231, SUM159, MCF10Ca1h) and ER^+^/PR^+ ^(T47D, MCF7, BT474). These cell lines were chosen for their well characterized phenotype, specifically to limit any confounding effects of uncharacterized primary breast cancer cells. Cells were overlaid onto one of the three matrices (adjusted for equal protein concentration) and allowed to respond to a chemoattractant. Four independent experiments, each with different pools of patient samples (minimum of three patient samples per pool), were performed with each individual experiment repeated in duplicate to ensure repeatability. Cell invasion through the control matrix was low for all cell lines tested (Figure 2a). Interestingly, the ability of cells to invade through the ECM was dependent upon both the cell's hormone receptor status and the source of the ECM. The ER^-^/PR^- ^cells were consistently more invasive when in contact with the AA ECM. Conversely, all of the ER^+^/PR^+ ^cell lines tested were more invasive when in contact with the CAU ECM, suggesting that the invasiveness of cells was enhanced by a hormone receptor-dependent mechanism in the presence of CAU ECM. The increased invasiveness may have occurred through a combinatorial effect of ECM components and hormones in the chemoattractant serum. This potential hormone receptor-dependent stimulus had no effect on the ER^-^/PR^- ^cells. In the AA-derived ECM, however, a hormone-independent mechanism appeared to further stimulate the invasiveness of the ER^-^/PR^- ^cells.

Soft agar transformation assays were performed to measure whether the addition of either ECM could differentially influence cell survival under anchorage-independent conditions, mimicking changes that occur during tumorigenesis. Results were similar to those observed with invasion assays; the AA ECM significantly increased the number of colonies for the ER^-^/PR^- ^MDA-MB-231 cells above all other treatments (*P *< 0.01, Figure 2b). Similar results were observed with SUM159 and MCF10Ca1h cells (Additional file 1, Figure S3). Additionally, the CAU ECM significantly increased colony formation above untreated controls for the ER^+^/PR^+ ^T47D cell line (*P *< 0.05 Figure 2b, similarly for MCF7, ZR75-1, and BT474 cells; Additional file 1, Figure S3). Thus, the ability of the cells to survive and proliferate in hostile, anchorage-independent conditions appeared dependent upon both the source of the ECM and the hormonal status of the cells.

To further analyze the composition of the whole breast ECM, we assessed its protease activity. The stroma can act as a reservoir for matrix metalloproteinases (MMPs), which degrade ECM proteins and process a number of bioactive molecules. In breast tissue, MMP activity contributes to epithelial cell death, influences tissue remodeling, and has been implicated in cancer invasion and metastasis [33-35]. Gelatin zymography was employed, and in multiple experiments the protease activity of at least two different MMPs was upregulated in the CAU compared to the AA ECM as determined using gelatin zymography (representative gel; Figure 2c). When compared to the fibroblast PCR array analysis, there was no significant increase in MMP expression in the CAU fibroblasts compared to the AA, indicating that cells other than fibroblasts produce MMPs, or that there is increased stimulation of MMP activity in CAU versus AA ECM.

### Effects of ECM on tumorigenicity *in vivo*

**Figure 3:**
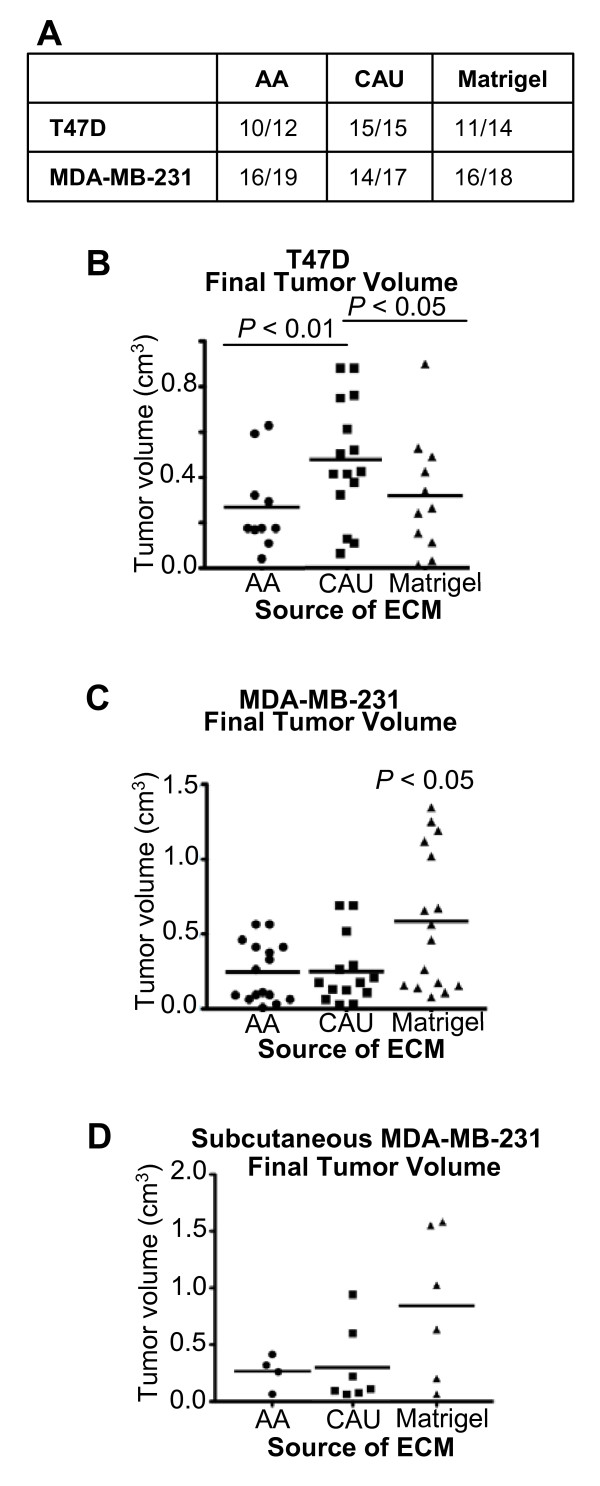
**Caucasian-American derived extracellular matrix molecules (ECM) enhances the tumorigenicity of estrogen receptor (ER)**^**+**^**/progesterone receptor (PR)^+ ^breast cancer cells**. **(a) **Indicates the number of tumors detected per animals injected. **(b,c) **Data represent mean ± SD of the final tumor volume of ER^+^/PR^+ ^(T47D) or ER^-^/PR^- ^(MDA-MB-231) breast cancer cells suspended in the indicated ECM and injected into the abdominal mammary gland. **(d) **Data represent mean ± SD of the final tumor volume of MDA-MB-231 breast cancer cells suspended in the indicated ECM and injected subcutaneously. AA = African-American, CAU = Caucasian-American.

We sought to address whether factors within the breast ECM, removed from the physical presence of stromal cells, could affect the tumorigenicity of breast cancer cells *in vivo*. As described by McDaniel *et al. *[26], ER^-^/PR^- ^MDA-MB-231 or ER^+^/PR^+ ^T47D breast cancer cells were mixed with control matrix (Matrigel), AA or CAU ECM (adjusted for equal protein content) and injected into the abdominal mammary fat pad of female, athymic nude mice. No significant difference in tumor efficiency was observed among the treatments (Figure 3a). However, the CAU ECM significantly increased tumor growth of the ER^+^/PR^+ ^T47D cells compared to either the AA ECM or control matrix control (Figure 3b; *P *< 0.01 and 0.05, respectively). AA ECM elicited no difference in growth compared to the control matrix. The resultant T47D tumors from all treatment groups were ER^+^; confirming that hormone status of the cells was retained during tumor formation (data not shown). Tumor morphology and percentage necrosis were similar between treatments. These data support the observation that factors within CAU ECM selectively interact with ER^+^/PR^+ ^cells to increase their aggressiveness. The *in vitro *data demonstrating enhanced MMP activity and increased cytokine production in the CAU microenvironment could potentially account for this increase in tumor growth.

Conversely, injecting ER^-^/PR^- ^MDA-MB-231 cells with ECM derived from either group gave no selective advantage in tumor growth. Only cells injected with growth factor-rich control matrix had significantly increased final tumor volume (*P *< 0.05, Figure 3c). To confirm that the ECM's effect on tumorigenicity was not masked due to interactions with the host mammary gland, MDA-MD-231 cells were mixed with control matrix, AA or CAU ECM, adjusted for equal protein content, and injected subcutaneously into athymic nude mice. A similar trend to results obtained from the orthotopic site was observed. Only cells injected with the control matrix had substantially larger final tumor volume compared to cells injected with ECM derived from either group, although no statistically significant difference was observed between either ECM (Figure 3d). No significant difference in tumor latency was observed among the treatments.

**Figure 4:**
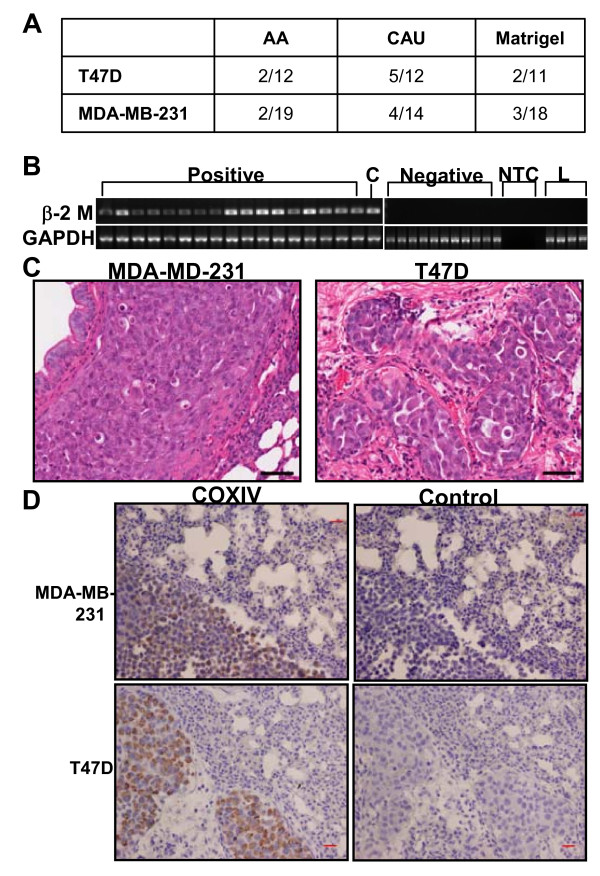
**Caucasian-American extracellular matrix molecules (ECM) enhances metastasis to lung**. **(a) **Number of animals positive for lung metastasis per animals injected. **(b) **Ethidium bromide stained gel of PCR products; RNA was analyzed by quantitative real-time PCR for lung metastasis using human-specific primers to β2 microglobulin. C = control human cDNA; L = mouse lung cDNA; NTC = no template control. **(c) **Representative hematoxylin and eosin (H&E)-stained mouse lungs with human breast cancer cell metastasis. **(d) **Immunohistochemical detection of metastasized cells with the human-specific antibody COXIV (left panels) or corresponding negative controls (right panels) in mouse lung. Scale bar = 50 μM. AA = African-American, CAU = Caucasian-American.

Metastasis to the lung and liver was low for all treatments and cell lines tested. Of the 75 lung samples analyzed, only 18 tested positive for lung metastasis via pathological examination, immunohistochemical analysis for human specific COXIV, and qRT-PCR for human β2-microglobin [26] (Figure 4). No liver metastases were found by histological examination. Regardless of the cell type injected, the CAU ECM elicited at least twice the metastases as AA ECM (Figure 4a). Figure 4c depicts representative images of hematoxylin and eosin (H&E)-stained lungs with overt metastases resulting from the two cell types, and metastatic cells within the lung were detected by a human specific COXIV antibody (Figure 4d). No correlation between the size of the primary tumor and metastasis and no significant difference between the amount of metastasis and treatment was observed for all samples analyzed.

### Mass spectrometry analysis of the ECM

**Figure 5:**
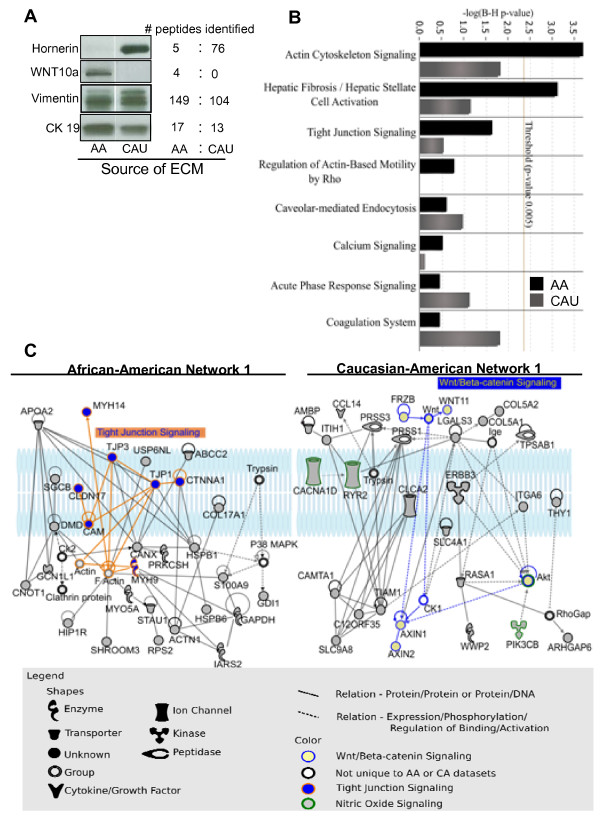
**Proteomic analysis of African-American and Caucasian-American breast extracellular matrix molecules (ECM)**. **(a) **Western blot of proteins identified by mass spectrometry with corresponding identified peptides. **(b) **Datasets of proteins unique to African-American or Caucasian-American ECM grouped according to significant association to canonical pathways. B-H = Benjamini-Hochberg. **(c) ***In silico *analysis of proteins: top signaling network of proteins unique to the African-American (left) or Caucasian-American (right) datasets. AA = African-American, CAU = Caucasian-American.

To determine whether the overall composition of the ECM differed between premenopausal AAs and CAUs, breast ECM proteins were intimately examined by LC-MS/MS. Three independent ECM extraction experiments, using different patient samples for each experiment, with at least three patients per group, (that is, a total of six individual ECM pools) were analyzed using LC-MS/MS. Among the 4,288 AA and 4,301 CAU proteins identified, only 1,759 were common between the groups. Ingenuity pathways analysis (IPA, Ingenuity Systems, http://www.ingenuity.com) was used for functional analysis of the results. The majority of the high abundance proteins (≥30 peptide matches) consisted of structural ECM proteins including numerous collagen species. These were comparatively similar for either group. Therefore, we excluded those identified by ≥30 peptides to focus on those with lower abundance. Additionally, although it is possible to identify a protein from a single peptide match after careful inspection of the fragment's pattern and other characteristics, we excluded these single unique peptides and focused our studies on those above two peptide matches. During analysis, proteins reported to be exclusively nuclear were excluded as cellular contamination (23.4% for the AA dataset and 23.0% for the CAU dataset). In addition to ECM structural proteins, another distinct feature of the microenvironment is the presence of extracellular matrix vesicles (MV) and exosomes, small sacs secreted from the cell surface which are enclosed by a membrane structurally similar to that of the plasma membrane [36]. Molecules located within MV/exosomes perform diverse functions outside of the cell including growth factor storage and secretion, immune regulation, ECM mineralization, and RNA shuttling. Interestingly, tumor-associated MV/exosomes have been shown to contribute to the ability of tumor cells to escape immune surveillance, degrade ECM to facilitate invasion, and stimulate angiogenesis [36]. Therefore, no cytosolic proteins that may have been released into the ECM by MV/exosomes were excluded. Representative western blot images of four randomly chosen proteins were selected for validation of LC-MS/MS analysis with their corresponding numbers of peptides identified (Figure 5a, representative spectra shown in Additional file 1, Figure S4).

Pathways analyses by IPA revealed that the actin cytoskeleton signaling and the hepatic fibrosis/hepatic stellate cell activation pathways were only significant for the AA dataset, based on a strict *P *value threshold of 0.005 and Benjamini-Hochberg correction (Figure 5b). Both of these pathways, as well as tight junction signaling and regulation of actin-based motility by Rho, had numerous molecules in common including Rho-associated kinase (ROCK), actin, and several myosins. The significant presence of these pathways in only the AA microenvironment implies unique cytoskeletal signaling compared to that in CAU.

To further examine differences among datasets, we analyzed networks created by proteins found exclusively in the AA or the CAU datasets. The network with the highest score for the AA dataset contained seven nodes representing proteins/genes associated with tight junction signaling and actin cytoskeleton signaling pathway (Figure 5c). The top network generated by the CAU dataset contained several other adhesion molecules and the epidermal growth factor receptor ErbB3, which is known to promote epithelial cell growth and antiapoptotic signaling. ErbB3 is often overexpressed in human breast cancers, frequently in conjunction with overexpression of the proto-oncogene ErbB2/HER2 [37]. Interestingly, the CAU dataset also contained molecules associated with the Wnt/β-catenin pathway (Figure 5c), whose dysregulation has been implicated in breast cancer development and progression [38]. Additional networks for the AA can CAU datasets can be found in Additional file 1, Figures S5a-d and S6a-d.

Based on annotations by two manually curated databases (IPA and BIOBASE, http://www.biobase-international.com), similar and unique proteins to each dataset, and their known associations with breast cancer, were examined. The AA and CAU datasets had 52 common molecules (Table 1) and 48 and 47 unique proteins, respectively (Tables 2 and 3). Further functional analyses of these proteins indicated that the AA dataset were mainly related to initiation events, including tumorigenesis and neoplasia. Alternatively, the proteins unique to the CAU dataset were primarily involved with tumor growth or metastasis.

**Table 1:** Proteins found in both African-American and Caucasian-American datasets reported to be involved in breast cancer, as annotated by Ingenuity Systems or BIOBASE.

Symbol	Entrez gene name	Location	Entrez gene ID
ABCC4	ATP-binding cassette, sub-family C (CFTR/MRP), member 4	Plasma membrane	10257
ACTA2	Actin, alpha 2, smooth muscle, aorta	Cytoplasm	59
ACTB	Actin, beta	Cytoplasm	60
ADAMTS15	ADAM metallopeptidase with thrombospondin type 1 motif, 15	Extracellular space	170689
ADRA1B	Adrenergic, alpha-1B-, receptor	Plasma membrane	147
ANXA2	Annexin A2	Plasma membrane	302
APOB	Apolipoprotein B (including Ag(x) antigen)	Extracellular space	338
APOD	Apolipoprotein D	Extracellular space	347
ATP5B	ATP synthase, H+ transporting, mitochondrial F1 complex, beta polypeptide	Cytoplasm	506
AXL	AXL receptor tyrosine kinase	Plasma membrane	558
C3	Complement component 3	Extracellular space	718
CAV1	Caveolin 1, caveolae protein, 22 kDa	Plasma membrane	857
CNR2	Cannabinoid receptor 2 (macrophage)	Plasma membrane	1269
COL18A1	Collagen, type XVIII, alpha 1	Extracellular space	80781
CYP2C9	Cytochrome P450, family 2, subfamily C, polypeptide 9	Cytoplasm	1559
DCD	Dermcidin	Extracellular space	117159
DSP	Desmoplakin	Plasma membrane	1832
FES	Feline sarcoma oncogene	Cytoplasm	2242
FN1	Fibronectin 1	Plasma membrane	2335
GART	Phosphoribosylglycinamide formyltransferase/synthetase,	Cytoplasm	2618
GFRA1	GDNF family receptor alpha 1	Plasma membrane	2674
HSPA5	Heat shock 70 kDa protein 5 (glucose-regulated protein, 78 kDa)	Cytoplasm	3309
HSPG2	Heparan sulfate proteoglycan 2	Plasma membrane	3339
IGF2R	Insulin-like growth factor 2 receptor	Plasma membrane	3482
IGKC	Immunoglobulin kappa constant	Extracellular space	3514
ITGA2	Integrin, alpha 2 (CD49B, alpha 2 subunit of VLA-2 receptor)	Plasma membrane	3673
JUP	Junction plakoglobin	Plasma membrane	3728
LAMA3	Laminin, alpha 3	Extracellular space	3909
LDHA	Lactate dehydrogenase A	Cytoplasm	3939
LEPR	Leptin receptor	Plasma membrane	3953
LGALS1	Lectin, galactoside-binding, soluble, 1	Extracellular space	3956
MAGED1	Melanoma antigen family D, 1	Plasma membrane	9500
MAP3K4	Mitogen-activated protein kinase kinase kinase 4	Cytoplasm	4216
MUC16	Mucin 16, cell surface associated	Extracellular space	94025
MUC5AC	Mucin 5AC, oligomeric mucus/gel-forming	Extracellular space	4586
NF1	Neurofibromin 1	Cytoplasm	4763
PCM1	Pericentriolar material 1	Cytoplasm	5108
POSTN	Periostin, osteoblast specific factor	Extracellular space	10631
PRDX3	Peroxiredoxin 3	Cytoplasm	10935
PRKCG	Protein kinase C, gamma	Cytoplasm	5582
PRLR	Prolactin receptor	Plasma membrane	5618
PTN	Pleiotrophin	Extracellular space	5764
PTPN13	Protein tyrosine phosphatase, non-receptor type 13	Cytoplasm	5783
PXDN	Peroxidasin homolog (Drosophila)	Unknown	7837
SERPINA1	Serpin peptidase inhibitor, clade A (alpha-1 antiproteinase, antitrypsin) 1	Extracellular space	5265
SLC16A3	Solute carrier family 16, member 3 (monocarboxylic acid transporter 4)	Plasma membrane	9123
SLC19A1	Solute carrier family 19 (folate transporter), member 1	Plasma membrane	6573
TES	Testis derived transcript (3 LIM domains)	Plasma membrane	26136
TNC	Tenascin C	Extracellular space	3371
TUBA1A	Tubulin, alpha 1a	Cytoplasm	7846
VCL	Vinculin	Plasma membrane	7414
VTN	Vitronectin	Extracellular space	7448

**Table 2:** Proteins found unique to the African-American dataset that are reported to be involved in breast cancer, as annotated by Ingenuity Systems or BIOBASE.

Symbol	Entrez gene name	Location	Entrez gene ID
ADAM12	ADAM metallopeptidase domain 12	Plasma membrane	8038
ADRA2C	Adrene gic, alpha-2C-, receptor	Plasma membrane	152
AKR1C1	Aldo-keto reductase family 1, member C1 (dihydrodiol dehydrogenase 1)	Cytoplasm	1645
ANK1	Ankyrin 1, erythrocytic	Plasma membrane	286
ANXA1	Annexin A1	Plasma membrane	301
ANXA5	Annexin A5	Plasma membrane	308
BCAS1	Breast carcinoma amplified sequence 1	Unknown	8537
CAMKK1	Calcium/calmodulin-dependent protein kinase kinase 1, alpha	Cytoplasm	84254
CANX	Calnexin	Cytoplasm	821
CASP1	Caspase 1, apoptosis-related cysteine peptidase (interleukin 1, convertase	Cytoplasm	834
CD69	CD69 molecule	Plasma membrane	969
CDH11	Cadherin 11, type 2, OB-cadherin (osteoblast)	Plasma membrane	1009
CDR2	Cerebellar degeneration-related protein 2, 62 kDa	Cytoplasm	1039
CTSD	Cathepsin D	Cytoplasm	1509
DAB2	Disabled homolog 2, mitogen-responsive phosphoprotein (Drosophila)	Plasma membrane	1601
DECR1	2,4-Dienoyl CoA reductase 1, mitochondrial	Cytoplasm	1666
DFNA5	Deafness, autosomal dominant 5	Unknown	1687
DLC1	Deleted in liver cancer 1	Cytoplasm	10395
EDNRB	Endothelin receptor type B	Plasma membrane	1910
EEF1A1	Eukaryotic translation elongation factor 1 alpha 1	Cytoplasm	1915
ERBB4	V-erb-a erythroblastic leukemia viral oncogene homolog 4 (avian)	Plasma membrane	2066
FBLN1	Fibulin 1	Extracellular space	2192
GAPDH	Glyceraldehyde-3-phosphate dehydrogenase	Cytoplasm	2597
GRIN2D	Glutamate receptor, ionotropic, *N*-methyl d-aspartate 2D	Plasma membrane	2906
GSN	Gelsolin (amyloidosis, Finnish type)	Extracellular space	2934
HSPB1	Heat shock 27 kDa protein 1	Cytoplasm	3315
IGF1R	Insulin-like growth factor 1 receptor	Plasma membrane	3480
KDR	Kinase insert domain receptor (a type III receptor tyrosine kinase)	Plasma membrane	3791
LTF	Lactotransferrin	Extracellular space	4057
MET	Met proto-oncogene (hepatocyte growth factor receptor)	Plasma membrane	4233
MMP14	Matrix metallopeptidase 14 (membrane-inserted)	Extracellular space	4323
MUC6	Mucin 6, oligomeric mucus/gel-forming	Extracellular space	4588
NLRP1	NLR family, pyrin domain containing 1	Cytoplasm	22861
ORM1	Orosomucoid 1	Extracellular space	5004
PDGFRB	Platelet-derived growth factor receptor, beta polypeptide	Plasma membrane	5159
PLXNA1	Plexin A1	Plasma membrane	5361
POR	P450 (cytochrome) oxidoreductase	Cytoplasm	5447
PTPRG	Protein tyrosine phosphatase, receptor type, G	Plasma membrane	5793
ROCK1	Rho-associated, coiled-coil containing protein kinase 1	Cytoplasm	6093
S100A9	S100 calcium binding protein A9	Cytoplasm	6280
SERPINA3	Serpin peptidase inhibitor, clade A (alpha-1 antiproteinase), member 3	Extracellular space	12
SERPINB5	Serpin peptidase inhibitor, clade B (ovalbumin), member 5	Extracellular space	5268
SOD1	Superoxide dismutase 1, soluble	Cytoplasm	6647
SOD2	Superoxide dismutase 2, mitochondrial	Cytoplasm	6648
SYK	Spleen tyrosine kinase	Cytoplasm	6850
TAGLN	Transgelin	Cytoplasm	6876
TJP1	Tight junction protein 1 (zona occludens 1)	Plasma membrane	7082
TXNRD1	Thioredoxin reductase 1	Cytoplasm	7296

**Table 3:** Proteins found unique to the Caucasian-American dataset that are reported to be involved in breast cancer, as annotated by Ingenuity Systems or BIOBASE.

Symbol	Entrez gene name	Location	Entrez gene ID
ABCC5	ATP-binding cassette, sub-family C (CFTR/MRP), member 5	Plasma membrane	10057
ACE	Angiotensin I converting enzyme (peptidyl-dipeptidase A) 1	Plasma membrane	1636
BCAR3	Breast cancer anti-estrogen resistance 3	Cytoplasm	8412
CDON	Cdon homolog (mouse)	Plasma membrane	50937
CLCA2	CLCA family member 2, chloride channel regulator	Plasma membrane	9635
CSF1R	Colony stimulating factor 1 receptor	Plasma membrane	1436
CTGF	Connective tissue growth factor	Extracellular space	1490
DAPK1	Death-associated protein kinase 1	Cytoplasm	1612
DLG5	Discs, large homolog 5 (Drosophila)	Plasma membrane	9231
ERBB3	V-erb-b2 erythroblastic leukemia viral oncogene homolog 3 (avian)	Plasma membrane	2065
FRZB	Frizzled-related protein	Extracellular space	2487
GREB1	GREB1 protein	Cytoplasm	9687
HGF	Hepatocyte growth factor (hepapoietin A; scatter factor)	Extracellular space	3082
HPN	Hepsin (transmembrane protease, serine 1)	Plasma membrane	3249
HSPA8	Heat shock 70 kDa protein 8	Cytoplasm	3312
IL17RB	Interleukin 17 receptor B	Plasma membrane	55540
IRS2	Insulin receptor substrate 2	Cytoplasm	8660
ITGA6	Integrin, alpha 6	Plasma membrane	3655
KCNH1	Potassium voltage-gated channel, subfamily H (eag-related), member 1	Plasma membrane	3756
KISS1	KiSS-1 metastasis-suppressor	Cytoplasm	3814
LGALS3	Lectin, galactoside-binding, soluble, 3	Extracellular space	3958
LPA	Lipoprotein, Lp(a)	Extracellular space	4018
LPHN2	Latrophilin 2	Plasma membrane	23266
LTA	Lymphotoxin alpha (TNF superfamily, member 1)	Extracellular space	4049
MAP2K5	Mitogen-activated protein kinase kinase 5	Cytoplasm	5607
MINK1	Misshapen-like kinase 1 (zebrafish)	Cytoplasm	50488
NOS3	Nitric oxide synthase 3 (endothelial cell)	Cytoplasm	4846
NOTCH4	Notch homolog 4 (Drosophila)	Plasma membrane	4855
NRG1	Neuregulin 1	Extracellular space	3084
OAS3	2'-5'-oligoadenylate synthetase 3, 100 kDa	Cytoplasm	4940
P4HB	Procollagen-proline, 2-oxoglutarate 4-dioxygenase (proline 4-hydroxylase), beta polypeptide	Cytoplasm	5034
PKD1	Polycystic kidney disease 1 (autosomal dominant)	Plasma membrane	5310
PLG	Plasminogen	Extracellular space	5340
PTPRF	Protein tyrosine phosphatase, receptor type, F	Plasma membrane	5792
RASA1	RAS p21 protein activator (GTPase activating protein) 1	Cytoplasm	5921
SERPINI2	Serpin peptidase inhibitor, clade I (pancpin), member 2	Extracellular space	5276
SH3RF1	SH3 domain containing ring finger 1	Cytoplasm	57630
SOCS1	Suppressor of cytokine signaling 1	Cytoplasm	8651
TBC1D9	TBC1 domain family, member 9 (with GRAM domain)	Plasma membrane	23158
TGFBR2	Transforming growth factor, beta receptor II (70/80 kDa)	Plasma membrane	7048
TIAM1	T-cell lymphoma invasion and metastasis 1	Cytoplasm	7074
TNN	Tenascin N	Plasma membrane	63923
TPI1	Triosephosphate isomerase 1	Cytoplasm	7167
VWF	von Willebrand factor	Extracellular space	7450
WISP2	WNT1 inducible signaling pathway protein 2	Extracellular space	8839
WNT11	Wingless-type MMTV integration site family, member 11	Extracellular space	7481
YWHAZ	Tyrosine 3-monooxygenase/tryptophan 5-monooxygenase activation protein, zeta polypeptide	Cytoplasm	7534

### Biologically active estrogen and androgen metabolites

**Figure 6:**
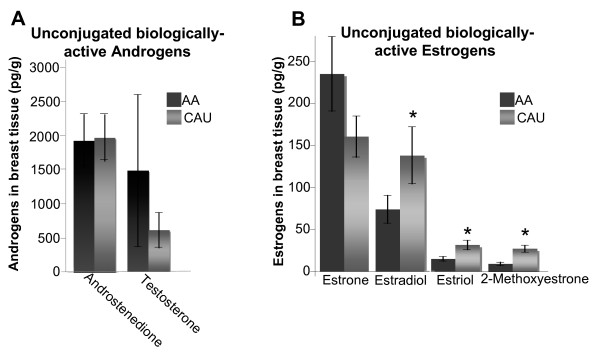
**Detection of estrogens and androgens in whole breast tissue**. Data represent the mean (n = 18 African-American (AA) and 20 Caucasian-American (CAU)) of biologically active androgens **(a) **or estrogens **(b) **± standard error analyzed via chromatography-tandem mass spectrometry. *P < 0.005; Wilcoxon matched pairs or paired t test, accordingly.

In this study, we show that CAU-derived ECM enhanced the aggressiveness of ER^+^/PR^+ ^breast cancer cells, suggesting the microenvironment from which the ECM was derived may have been exposed to a different hormonal milieu compared to the AA. Therefore, the hormones present in the breast microenvironment were quantitatively measured by a novel method of LC-MS/MS that simultaneously extracted and analyzed biologically active estrogen and androgen metabolites present in whole tissue samples. Breast tissues from fasting, premenopausal AA and CAU women (median age 32 and 31, respectively; n = 19 AA and 20 CAU) were analyzed. Both androstenedione and testosterone were detected in all tissues (Figure 6a, Table 4). Testosterone appeared elevated in the AA breast tissue; however, this observation was not statistically significant. It is of note that androgens levels were significantly higher in breast tissue compared to estrogens (*P *< 0.001, Tables 4 and 5), similar to what has been repeatedly observed in blood. More importantly, these results shows that the unconjugated biologically active estrogens found in premenopausal breast tissues were higher than those reported in blood [39]. These results warrant further study given the role of estrogen metabolites in tumorigenesis.

**Table 4:** Unconjugated biologically active androgens (pg/g) detected in breast tissue.

Sample ID	Wet weight (g)	Androstenedione	Testosterone	Total
AA1	0.307	2,546.9	428.5	2,975.4
AA2	0.293	2,393.9	422.5	2,816.4
AA3	0.305	673.7	20,433.2	21,106.9
AA4	0.290	2,922.5	628.7	3,551.2
AA6	0.375	1,196.0	419.9	1,615.9
AA7	0.317	6,634.4	872.9	7,507.3
AA8	0.324	443.1	152.6	595.7
AA10	0.272	2,748.0	470.5	3,218.6
AA11	0.249	844.5	224.6	1,069.2
AA12	0.270	1,099.8	184.6	1,284.4
AA13	0.269	601.8	120.1	721.9
AA14	0.289	2,890.2	373.8	3,264.0
AA15	0.299	4,678.6	664.0	5,342.6
AA16	0.345	832.0	184.3	1,016.3
AA17	0.298	1,314.9	203.8	1,518.7
AA18	0.299	562.4	667.5	1,229.9
AA19	0.263	910.0	184.4	1,094.4
AA5	0.348	1,588.9	227.4	1,816.3
Mean	0.303	1,937.9	1,492.4	3,430.3
SD	0.033	1,634.7	4,731.9	4,756.7
SEM	0.008	385.3	1,115.3	1,121.2
CA1	0.267	5,927.3	1,103.9	7,031.2
CA2	0.283	1,904.7	178.2	2,082.9
CA3	0.268	2,390.2	424.8	2,815.0
CA4	0.328	311.5	141.4	452.9
CA5	0.260	879.7	740.8	1,620.5
CA6	0.294	962.5	211.5	1,173.9
CA7	0.295	1,425.8	155.2	1,581.0
CA8	0.260	515.2	180.6	695.8
CA9	0.255	1,993.1	536.8	2,529.9
CA10	0.324	710.3	588.4	1,298.7
CA12	0.305	1,448.6	284.1	1,732.7
CA14	0.208	1,540.4	361.3	1,901.8
CA15	0.258	2,997.8	437.5	3,435.3
CA16	0.319	1,068.2	4,855.9	5,924.1
CA17	0.310	1,062.6	295.0	1,357.6
CA18	0.297	3,515.1	343.6	3,858.7
CA19	0.323	3,906.8	165.4	4,072.2
CA20	0.312	3,070.7	95.9	3,166.6
Mean	0.29	1,979.47	616.69	2,596.16
SD	0.03	1,440.86	1,087.75	1,757.64
SEM	0.01	339.61	256.38	414.28

SEM = standard error of the mean.

**Table 5:** Unconjugated biologically active estrogens (pg/g) detected in breast tissue.

Sample ID	Wet weight (g)	16KE2	E3	16aE1	16epiE3	17epiE3	3ME1	2ME1	4ME1	2ME2	E1	4ME2	E2	2OHE1	2OHE2	4OHE1	Total (pg/g)
AA1	0.307	ND	3.92	ND	ND	ND	ND	24.34	ND	ND	459.21	ND	138.15	ND	ND	ND	625.63
AA2	0.293	ND	6.86	ND	ND	ND	ND	4.21	ND	ND	456.38	ND	210.90	ND	21.41	ND	699.77
AA3	0.305	ND	6.44	ND	ND	ND	ND	4.33	ND	ND	26.35	ND	15.42	ND	ND	ND	52.55
AA4	0.290	ND	7.33	ND	ND	ND	ND	4.23	ND	ND	314.41	ND	105.14	ND	14.77	ND	445.87
AA5	0.348	ND	24.88	ND	ND	ND	ND	16.47	ND	ND	313.31	ND	69.34	ND	ND	ND	424.00
AA6	0.375	ND	18.97	ND	ND	ND	ND	NF	ND	ND	106.18	ND	34.39	ND	ND	ND	159.54
AA7	0.317	ND	43.72	ND	ND	ND	ND	5.49	ND	ND	728.23	ND	243.73	ND	ND	ND	1,021.17
AA8	0.324	ND	4.38	ND	ND	ND	ND	14.50	ND	ND	248.02	ND	45.20	ND	ND	ND	312.10
AA10	0.272	ND	11.02	ND	ND	ND	ND	NF	ND	ND	284.35	ND	103.53	ND	ND	ND	398.89
AA11	0.249	ND	16.88	ND	ND	ND	ND	5.28	ND	ND	70.04	ND	44.63	ND	ND	ND	136.83
AA12	0.270	ND	16.01	ND	ND	ND	ND	22.83	ND	ND	248.26	ND	38.55	ND	ND	ND	325.65
AA13	0.269	ND	16.53	ND	ND	ND	ND	4.26	ND	ND	20.14	ND	4.47	ND	ND	ND	45.41
AA14	0.289	ND	28.49	ND	ND	ND	ND	1.30	ND	ND	210.22	ND	59.68	ND	ND	ND	299.68
AA15	0.299	ND	4.07	ND	ND	ND	ND	20.11	ND	ND	351.99	ND	141.77	ND	ND	ND	517.93
AA16	0.345	ND	6.28	ND	ND	ND	ND	1.69	ND	ND	44.37	ND	19.59	ND	ND	ND	71.94
AA17	0.298	ND	33.88	ND	ND	ND	ND	14.32	ND	ND	135.21	ND	17.07	ND	ND	ND	200.47
AA18	0.299	ND	19.29	ND	ND	ND	ND	2.69	ND	ND	34.00	ND	1.21	ND	ND	ND	57.19
AA19	0.263	ND	2.51	ND	ND	ND	ND	5.32	ND	ND	171.89	ND	36.87	ND	ND	ND	216.60
Mean	0.30		15.08					9.46			234.59		73.87		18.09		333.96
SD	0.03		11.64					7.93			187.28		70.29		4.69		261.24
SEM	0.01		2.74					1.87			44.14		16.57		1.11		61.57
CA1	0.267	ND	39.42	ND	ND	ND	ND	39.50	ND	ND	350.35	ND	120.01	ND	ND	ND	549.27
CA2	0.283	ND	16.31	ND	ND	ND	ND	5.35	ND	ND	118.71	ND	385.77	ND	ND	ND	526.13
CA3	0.268	ND	37.05	ND	ND	ND	ND	44.14	ND	ND	131.34	ND	146.55	ND	ND	ND	359.08
CA4	0.328	ND	12.71	ND	ND	ND	ND	38.10	ND	ND	221.08	ND	615.33	ND	ND	ND	887.22
CA5	0.260	ND	6.87	ND	ND	ND	ND	27.61	ND	ND	50.04	ND	100.53	ND	ND	ND	185.05
CA6	0.294	ND	22.91	ND	ND	ND	ND	23.82	ND	ND	85.70	ND	36.74	ND	19.25	ND	188.41
CA7	0.295	ND	49.19	ND	ND	ND	ND	NF	ND	ND	98.10	ND	32.98	ND	40.37	ND	220.63
CA8	0.260	ND	23.83	ND	ND	ND	ND	10.86	ND	ND	34.00	ND	128.15	ND	ND	ND	196.85
CA9	0.255	ND	26.86	ND	ND	ND	ND	54.25	ND	ND	266.36	ND	153.19	ND	ND	ND	500.66
CA10	0.324	ND	15.30	ND	ND	ND	ND	10.95	ND	ND	61.18	ND	79.33	ND	ND	ND	166.76
CA12	0.305	ND	45.37	ND	ND	ND	ND	16.33	ND	ND	158.05	ND	106.95	ND	ND	ND	326.70
CA14	0.208	ND	67.15	ND	ND	ND	ND	25.67	ND	ND	337.20	ND	106.82	ND	ND	ND	536.84
CA15	0.258	ND	25.99	ND	ND	ND	ND	31.21	ND	ND	167.88	ND	64.90	ND	ND	ND	289.98
CA16	0.319	ND	32.34	ND	ND	ND	ND	26.20	ND	ND	351.61	ND	163.97	ND	ND	ND	574.12
CA17	0.310	ND	17.61	ND	ND	ND	ND	5.73	ND	ND	135.22	ND	69.24	ND	ND	ND	227.80
CA18	0.297	ND	101.07	ND	ND	ND	ND	63.23	ND	ND	174.84	ND	66.20	ND	ND	ND	405.34
CA19	0.323	ND	9.35	ND	ND	ND	ND	17.33	ND	ND	63.77	ND	46.59	ND	ND	ND	137.03
CA20	0.312	ND	19.01	ND	ND	ND	ND	26.06	ND	ND	78.28	ND	57.49	ND	ND	ND	180.83
Mean	0.29		31.57					27.43			160.21		137.82		29.81		358.82
SD	0.03		23.25					16.40			104.55		143.23		14.94		201.33
SEM	0.01		5.48					3.87			24.64		33.76		3.52		47.45

ND = not detected; SEM = standard error of the mean.

Of the 15 estrogen metabolites measured, only 5 were readily detectable as intrinsic components of breast tissue: estrone, estradiol, estriol, 2-methoxyestrone, and 2-hydroxyestradiol (Table 5). Estradiol, estriol and 2-methoxyestrone were significantly higher in the CAU breast tissue (*P *< 0.005), which was unanticipated given that previous studies have shown that premenopausal AA women have higher plasma concentrations of estrogens [40,41]. Additionally, tissue levels of 2-hydroxyestradiol, a known carcinogen, were also detected in two patient samples, one from each group (Table 5, mean pg/g = 18.1 ± 4.7 for AA and 29.8 ± 14.9 for CAU). The data presented here advocate for a more intensive analysis of the hormonal milieu of the breast microenvironment in addition to plasma levels, since local hormones are directly responsible for mediating cellular function and influence tumorigenesis.

## Discussion

This report is the first to analyze differences in the normal breast microenvironment of premenopausal women, and to show fundamental differences in the ability of breast ECM to influence the aggressiveness and tumorigenicity of breast cancer cells. The comprehensive LC-MS/MS identification of whole tissue hormone metabolites, as well as unique ECM proteins between the AA and CAU women, offers a novel insight into the intricacy of the breast microenvironment.

One limitation of this study, which must be addressed, is the lack of descriptive clinical data on the breast tissue isolated from the reduction mammoplasty patients. The tissue collected for fibroblast and whole breast tissue ECM isolation were considered pathological medical waste; therefore, informative clinical data including parity, body mass index, breast density, oral contraceptive use, phase of menstrual cycle were not available. Whether these important factors potentially had confounding effects on the observed results is regrettably unknown. In attempts to limit these effects, each experiment contained multiple replicates, and was repeated using as many different pools of patient samples feasible. A total of 50 CAU and 53 AA samples were used in the different analyses. However, the possibility remains that inherent factors from the tissue source could remain. Furthermore, we obtained samples from southern, eastern, and midwestern US, which may help eliminate the effects of socioeconomic factors if the samples had been obtained from one small geographical region. Determining whether clinical factors, or genetics, or a combination of the two, systematically relate as to why AAs develop a more aggressive cancer is not the purpose of this study. The objective of this study was not to determine how these discrepancies develop, but rather to use the information obtained to study their influence on breast cancer behavior. In addition, patient samples were pooled in order to obtain sufficient amount of ECM to perform these experiments. Although pooled samples are not ideal, a consistent pattern was observed with all results obtained even in this potentially confounding situation. Since no patient sample could be used twice, this suggests that our conclusions were not skewed by any single sample. Future studies to determine specific components in ECM responsible for these effects will require examination of individual tissues, if enough material becomes available from a single patient. Overall, the results presented provide valuable data for further investigation into the role of the microenvironment in cancer disparities, and potentially as a basis for future studies investigating factors such as parity and phase of menstrual cycle on breast cancer cell behavior.

Collectively, the data presented in this report suggest that the AA breast microenvironment is less permissive of tumor growth compared to the CAU breast microenvironment. Therefore, it is not surprising that only the more aggressive cells are able to survive and proliferate unrestrictedly in the suppressive microenvironment of AA breast tissue. The comparatively suppressive effects of the AA ECM may arise from both a physical restriction due to the types of structural material present in the ECM, and chemically from the signals present, or absent, in the microenvironment. Numerous reports have indicated that the spatial organization and composition of the ECM influence mammary cell behavior, and that alterations in ECM receptor expression facilitate malignant transformation [42].

The premenopausal stroma is not a static compartment; proliferation in the breast varies with the menstrual cycle, which requires the expansion and deposition of new ECM [43]. Increased deposition of molecules such as collagen can alter the ECM biophysical properties and increase extracellular cellular tension. ECM composition and rigidity modulate cell-ECM interactions and have a significant impact on cell functions. Indeed, mammary epithelial cells cultured on matrices with increased stiffness have disrupted cell-cell junctions, increased proliferation, perturbed endogenous basement membrane assembly, and a dedifferentiated phenotype [44,45]. The development of breast cancer is characterized by the loss of tissue organization and an increase in tissue rigidity, suggesting that aberrant tension may facilitate the acquisition of a malignant phenotype [45]. For example, primary mammary epithelial cells cultured on floating collagen gels were shown to differentiate in response to lactogenic hormones only when plated on collagen gels with reduced tensional forces. When plated on gels with increased tension, the extracellular forces promoted cell spreading, increased MMP activity, and inhibited acini formation and cellular differentiation [46]. Interestingly, triple-negative tumors (ER^-^/PR^- ^and lacking HER2 amplification) are composed of undifferentiated cells, potentially resulting from a small, localized area of matrix stiffness and high tension. Paszek *et al. *demonstrated that matrix stiffness promotes tumor-like behavior in mammary cells, and blocking integrin-dependent cell contractility reverted the malignant phenotype in culture [44]. Thus, if the premenopausal AA microenvironment is comparatively more restrictive in its composition/organization, as our data suggests, this may predispose AAs to triple-negative breast cancer. Further studies on this topic are warranted.

The use of a selective pressure to isolate a more tumorigenic cell is often used in studies seeking to identify the progenitor tumor-initiating cells (cancer stem cell) via culturing the cells in non-adherent conditions [47]. The rationale driving this culture system is that only the progenitor tumor-initiating cells are able to survive and self-renew when contact with the ECM is disrupted, whereas differentiated, non-tumor initiating cells experience anoikis and die [48]. Potentially a similar mechanism of selective pressure is actively selecting for the more aggressive cancer cell in the restrictive premenopausal AA microenvironment.

It is noteworthy that the invasiveness, tumorigenicity, and metastases of the ER^+^/PR^+ ^cells were enhanced in the presence of the CAU ECM. It has been similarly shown that breast cancer cell proliferation, in response to androgens, was dependent upon both the ER status of the cell and signals from the ECM. Specifically, ER^+ ^MCF7 cells proliferated in the presence of dihydrotestosterone (DHT) by an ERα-dependent mechanism; however, MDA-MB-231 cells responded to DHT by an ER-independent, αvβ3 integrin pathway [49]. Additional estrogen and ECM/integrin interactions related to tumorigenesis have been reported. Hypoxia-inducible factor 1α (HIF-1α), a transcription factor which is overexpressed in the majority of human carcinomas and controls central metastasis-associated pathways, was shown to increase anchorage-independent growth by downregulation of the α5 integrin [50]. Anchorage independent growth and decreased α5 integrin levels were reverted by treatment with the estrogen metabolite, 2-methoxyestradiol, a known pharmacological inhibitor of HIF-1α.

This is the first report to simultaneously analyze the biologically-active estrogens and androgens from each patient in whole breast tissue via LC-MS/MS; previous studies measured blood and urine levels. This method offers a more intimate analysis of the local hormone milieu of the breast microenvironment, compared to measuring circulating levels of hormones. Indeed, it is now well known that the local synthesis from the stromal cells dramatically contributes to the growth, function, and tumorigenesis of ER/PR positive and negative breast cells [51-53]. BRCA1 tumors, the majority of which are ER^-^, have been effectively prevented by ovariectomy [54]. Furthermore, it is proposed that the increased risk of breast cancer following pregnancy is due to high levels of estrogen and other pregnancy associated hormones that promote the growth of already initiated target cell populations [55]. Interestingly, the majority of breast cancers that develop during this time are ER^-^/PR^- ^suggesting that hormones affect the local microenvironment.

Different estrogen metabolites have been reported to act as either carcinogens or to protect from tumorigenesis, although their precise mechanisms are yet to be fully defined. The production of 16α-hydroxyestrone has been hypothesized to initiate breast cell transformation by acting as an estrogen agonist, increasing cellular proliferation and generating reactive oxygen species thereby causing DNA damage [56]. Conversely, 2-hydroxyestradiol has been shown to possess estrogen antagonist properties *in vivo *[56]. In this report, the primary estradiol/estrone metabolites detected were estriol, a product of the 16α-hydroxylation pathway, and 2-methoxyestrone, a product of the 2-hydroxylation pathway. It is of note that these two metabolites appear to be equally balanced in the tissue, as it has been shown that alterations in the ratio of C2/C16 estradiol/estrone hydroxylation can lead to anchorage-independent growth and tumorigenesis [57]. Analysis of these hormone metabolites in breast tissue, as opposed to circulating levels, could potentially be used for early detection of breast cancer in high-risk patients.

Our understanding of the interactions between the numerous cell types within breast, especially during tumorigenesis, still remains vague owing to the complexity of physical and chemical communication, and inherent differences between patients and their resultant types of cancer. However, apart from individual patient differences, there is indisputable evidence that breast carcinomas in premenopausal AA women tend to be triple negative and highly metastatic compared to breast carcinomas in CAU women. Identifying the initiating factors in the development of triple-negative breast cancer in premenopausal AA women will fill a gap of knowledge in breast cancer research. Why these women should have an increased incidence of this disease compared to other racial groups remains elusive.

## Conclusions

This report details the importance of the normal breast microenvironment on breast cancer cell behavior, an essential step in the investigation of the etiology of breast cancer. The data presented characterize the composition of normal premenopausal breast tissue and provides data for further investigation into the role of the microenvironment in cancer disparities.

## Competing interests

The authors declare that they have no competing interests.

## Authors' contributions

BKV and JMF conceived the project and designed all experiments. JMF performed all experiments and wrote the manuscript in consultation with BKV. TCM assisted in animal studies and qRT-PCR. MQ performed IPA and proteomic computational analyses. ZX and XX developed and performed MS procedures. MJM performed FACS. EG edited the manuscript and assisted in experiments. TDV assisted in the design and method of all MS procedures. All authors contributed to the analysis of data.

## Pre-publication history

The pre-publication history for this paper can be accessed here:

http://www.biomedcentral.com/1741-7015/8/27/prepub

## Supplementary Material

Additional file 1 **Supplementary table and figures**. Table S1: Real-time PCR primer sequences. Figure S1. Tumor characterization. **(a,b) **Humanized murine mammary glands, injected with primary metastatic breast cancer cells were stained for Ki67 **(a)**. Bar = 200 μm. Percentage proliferation: number of positive per total live cells in a minimum of three fields. **(b) **Representative hematoxylin and eosin (H&E)-stained section. Bar = 2,000 μm. Overt necrosis was determined using AxioVision Imaging software version 4.8. Data represent mean ± standard error. AA = African-American, CAU = Caucasian-American. Figure S2. Validation of qPCR arrays. Breast fibroblasts pools, derived from a minimum of three age-matched patients, were analyzed per array. Graphs represent fold increase of differentially regulated genes. Figure S3. Soft agar growth assay. Cell lines were assayed and stained with nitrobluetetrazolium before counting. Representative images of a minimum of two experiments per cell line. Bar = 200 μm. Figure S4. Representative MS/MS spectra of peptides identified from breast tissue ECM proteins. A = hornerin; B = wnt10a; C = vimentin; D = cytokeratin 19. Figure S5. *In silico *analysis in African-American extracellular matrix protein molecules. **(a-d) **Top signaling network of unique proteins. Mapped identifiers (shown in gray) were overlaid onto a global molecular network developed from information contained in the Ingenuity knowledge base. Networks were then algorithmically generated based on their connectivity and incorporated with other molecules with high connectivity (white). Nodes associated with breast cancer according to the IPA knowledge base, are utlined in yellow. Figure S6. *In silico *analysis in Caucasian-American extracellular matrix protein molecules. **(a-d) **As for Figure S5. Click here for file
