# Supplementary material for: The normal breast microenvironment of premenopausal women differentially influences the behavior of breast cancer cells in vitro and in vivo
Source: BMC Med. 2010 May 21;8:27. doi: 10.1186/1741-7015-8-27 (PMC2894739; doi:10.1186/1741-7015-8-27)
Supplement: Additional file 1 — Supplementary table and figures. Table S1: Real-time PCR primer sequences. Figure S1. Tumor characterization. (a,b) Humanized murine mammary glands, injected with primary metastatic breast cancer cells were stained for Ki67 (a). Bar = 200 μm. Percentage proliferation: number of positive per total live cells in a minimum of three fields. (b) Representative hematoxylin and eosin (H&E)-stained section. Bar = 2,000 μm. Overt necrosis was determined using AxioVision Imaging software version 4.8. Data represent mean ± standard error. AA = African-American, CAU = Caucasian-American. Figure S2. Validation of qPCR arrays. Breast fibroblasts pools, derived from a minimum of three age-matched patients, were analyzed per array. Graphs represent fold increase of differentially regulated genes. Figure S3. Soft agar growth assay. Cell lines were assayed and stained with nitrobluetetrazolium before counting. Representative images of a minimum of two experiments per cell line. Bar = 200 μm. Figure S4. Representative MS/MS spectra of peptides identified from breast tissue ECM proteins. A = hornerin; B = wnt10a; C = vimentin; D = cytokeratin 19. Figure S5. In silico analysis in African-American extracellular matrix protein molecules. (a-d) Top signaling network of unique proteins. Mapped identifiers (shown in gray) were overlaid onto a global molecular network developed from information contained in the Ingenuity knowledge base. Networks were then algorithmically generated based on their connectivity and incorporated with other molecules with high connectivity (white). Nodes associated with breast cancer according to the IPA knowledge base, are utlined in yellow. Figure S6. In silico analysis in Caucasian-American extracellular matrix protein molecules. (a-d) As for Figure S5. [file 1741-7015-8-27-S1.PPT]

## Slide 1
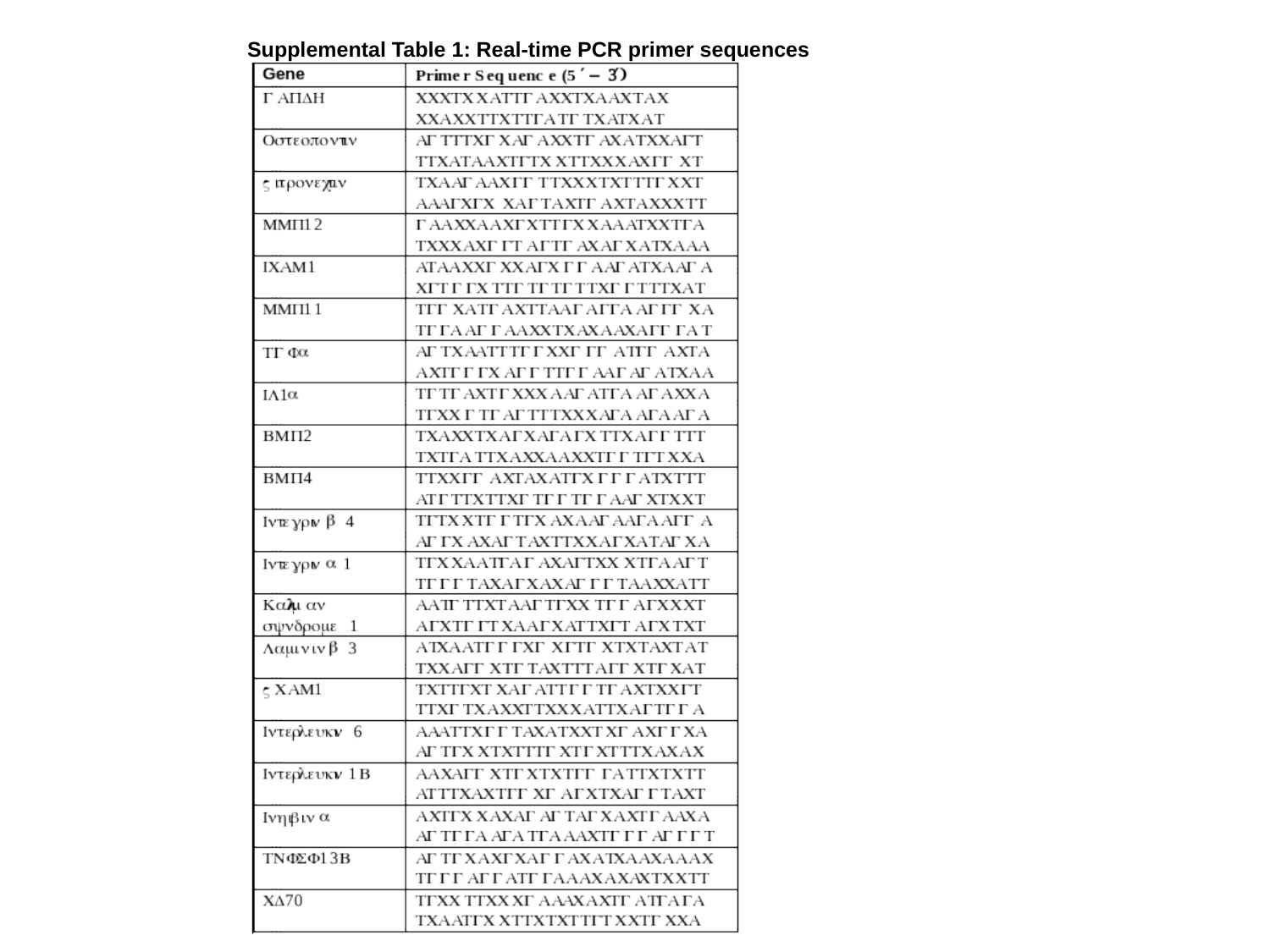

Supplemental Table 1: Real-time PCR primer sequences

## Slide 2
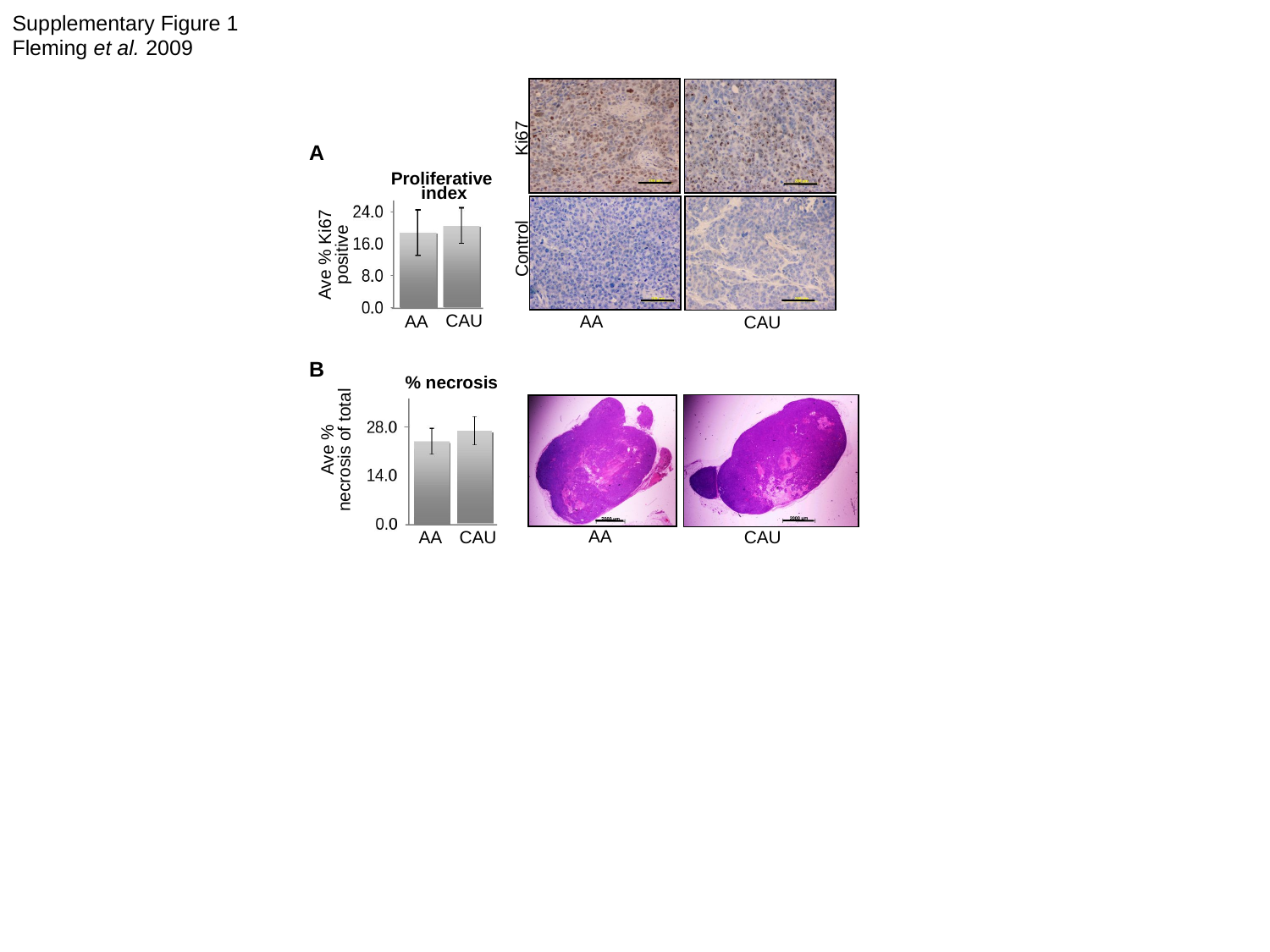

Supplementary Figure 1
Fleming et al. 2009
Ki67
A
Proliferative
index
Ave % Ki67
positive
Control
CAU
AA
AA
CAU
B
% necrosis
Ave %
necrosis of total
AA
CAU
CAU
AA

## Slide 3
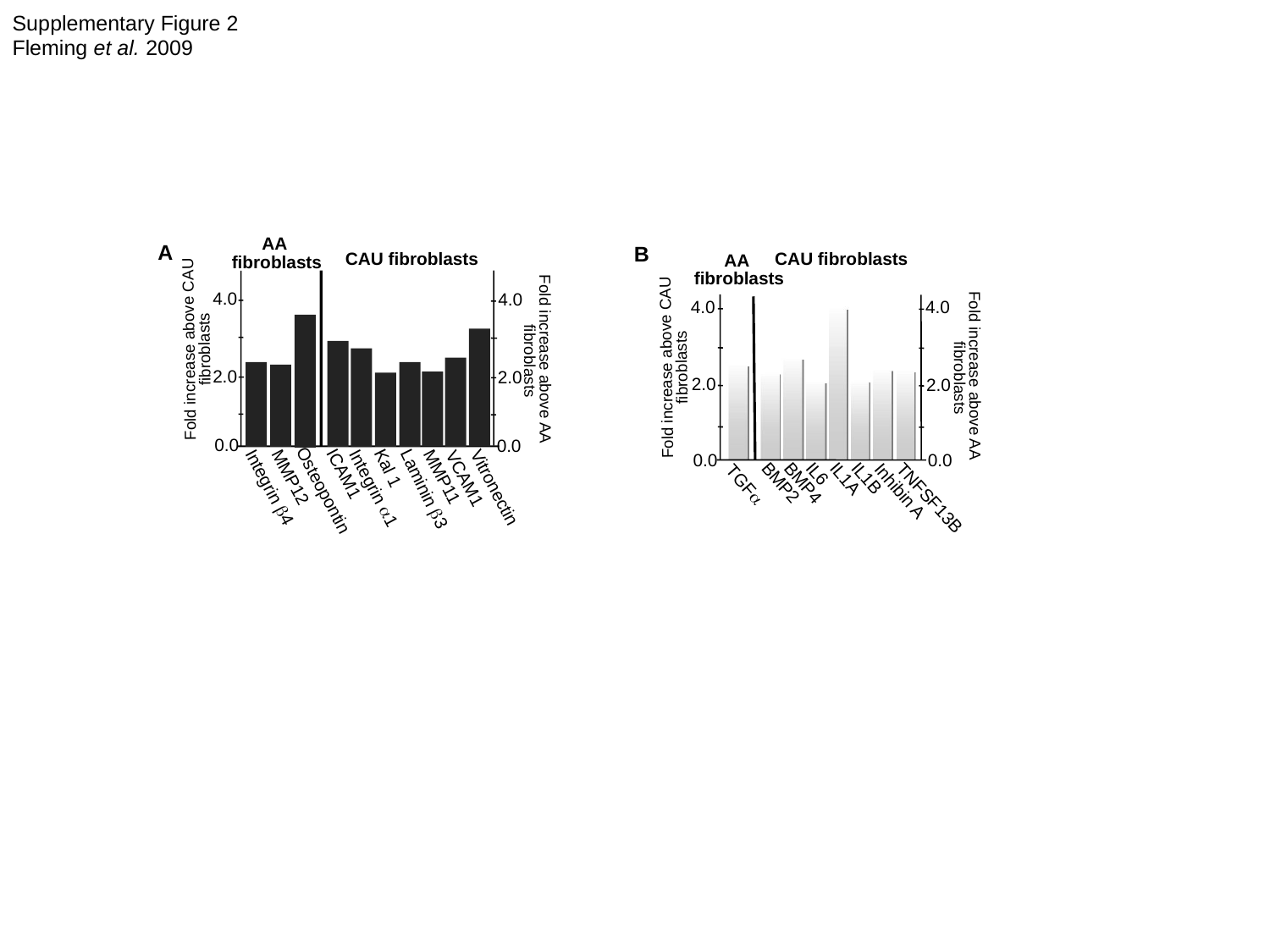

Supplementary Figure 2
Fleming et al. 2009
A
B
AA
fibroblasts
AA
fibroblasts
CAU fibroblasts
CAU fibroblasts
4.0
4.0
4.0
4.0
2.0
0.0
Fold increase above CAU
fibroblasts
Fold increase above CAU
fibroblasts
Fold increase above AA
fibroblasts
Fold increase above AA
 fibroblasts
2.0
2.0
2.0
0.0
0.0
0.0
Kal 1
ICAM1
IL6
MMP11
MMP12
VCAM1
IL1B
IL1A
BMP2
BMP4
TGFα
Integrin β4
Vitronectin
Laminin β3
Integrin α1
Inhibin A
Osteopontin
TNFSF13B

## Slide 4
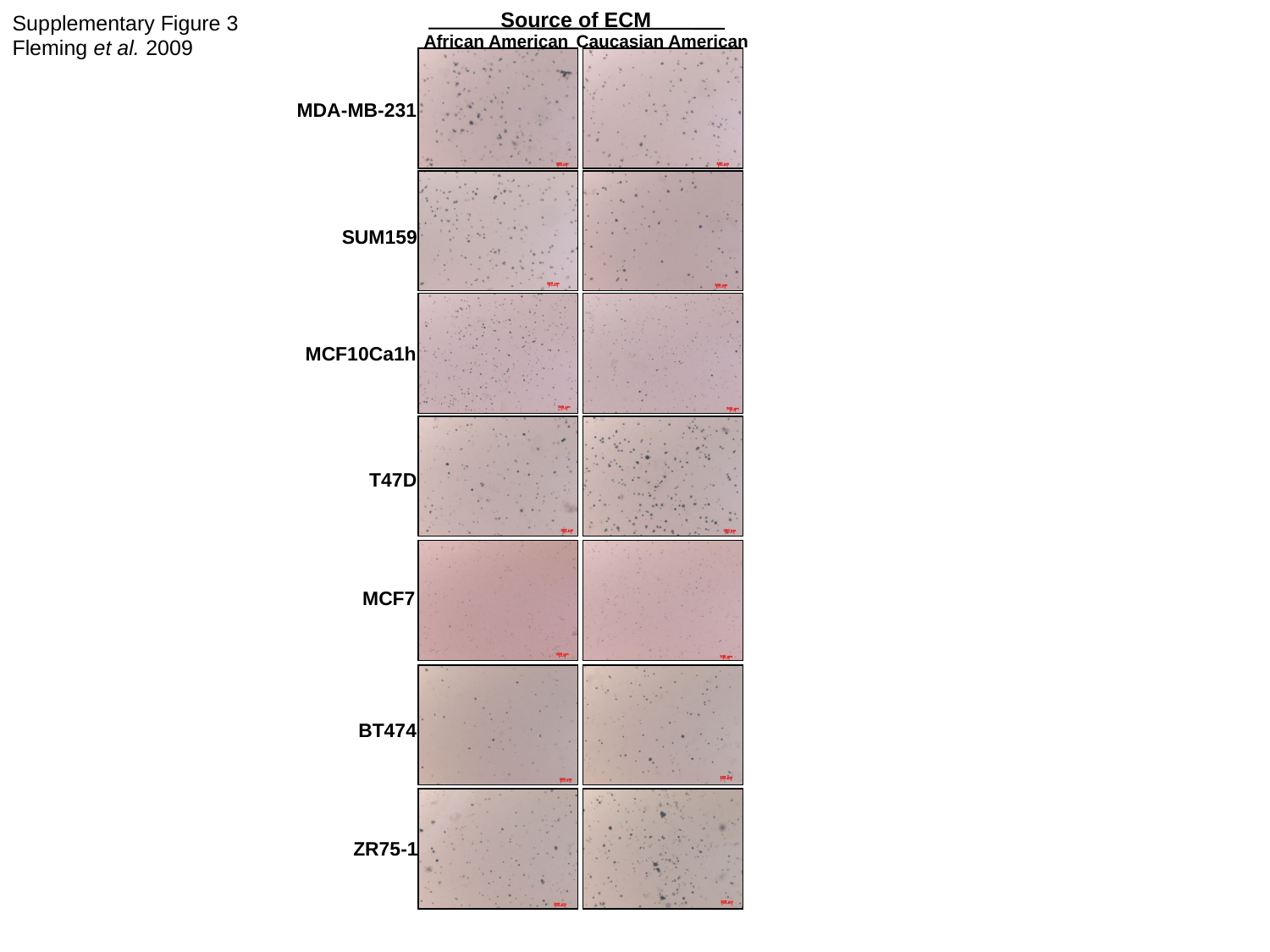

Source of ECM
African American
Caucasian American
MDA-MB-231
SUM159
MCF10Ca1h
T47D
 MCF7
BT474
ZR75-1
Supplementary Figure 3
Fleming et al. 2009

## Slide 5
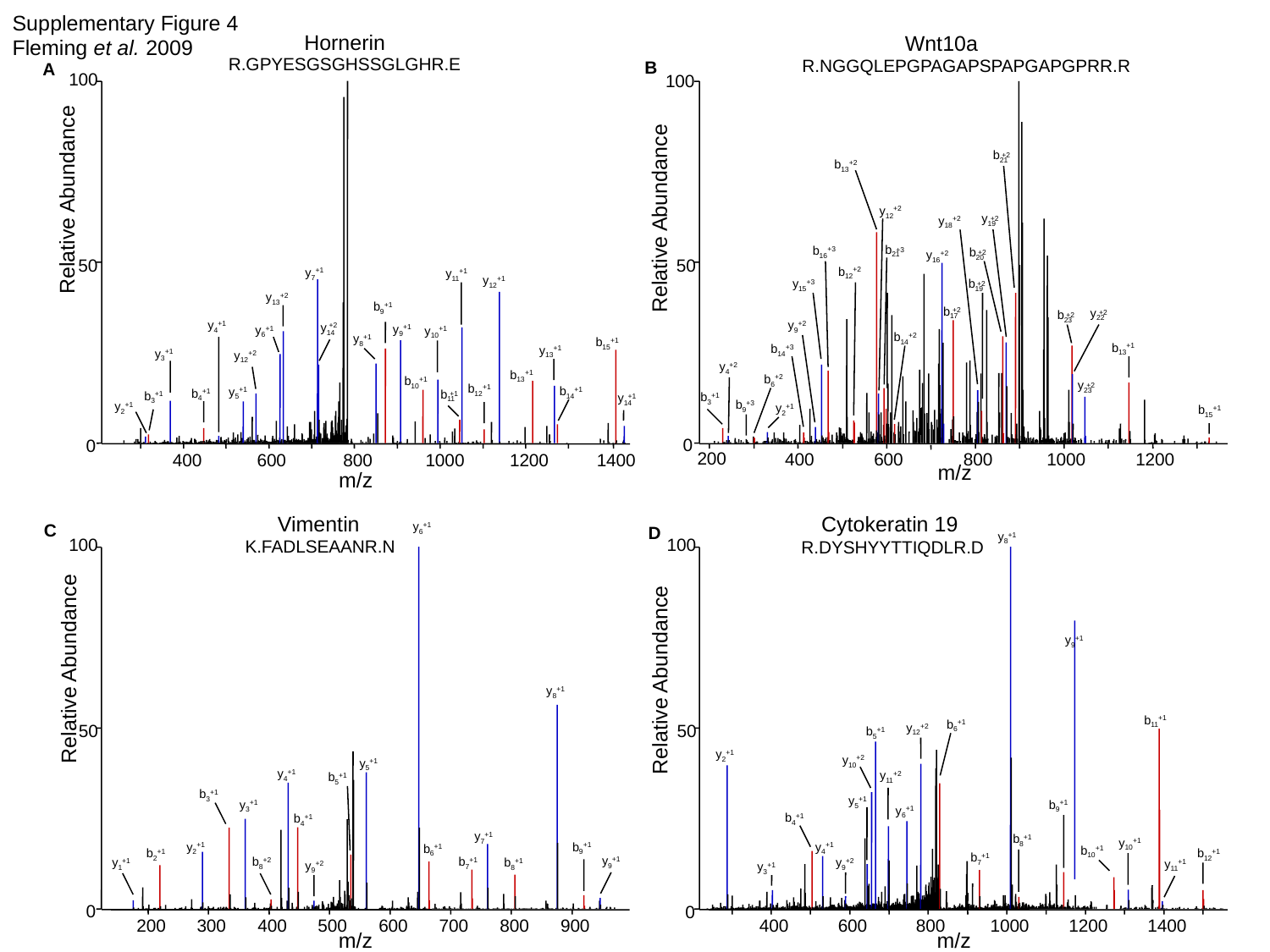

Supplementary Figure 4
Fleming et al. 2009
Hornerin
Wnt10a
R.NGGQLEPGPAGAPSPAPGAPGPRR.R
B
100
b21
+2
b13+2
y12+2
Relative Abundance
y19
+2
y18+2
b21
b16+3
+3
b20
+2
y16+2
50
b12+2
y15+3
b19
+2
b17
+2
y22
b23
+2
+2
y9+2
b14+2
b13+1
b14+3
y4+2
b6+2
y23
+2
b3+1
b9+3
y2+1
b15+1
0
200
400
600
800
1000
1200
m/z
R.GPYESGSGHSSGLGHR.E
A
100
Relative Abundance
50
y7+1
y11+1
y12+1
y13+2
b9+1
y4+1
+2
y14
y9+1
y6+1
y10+1
y8+1
b15+1
y13+1
y3+1
y12+2
b13+1
b10+1
b12+1
b14+1
y5+1
b4+1
b11
b3+1
+1
y14
+1
y2+1
0
400
600
800
1000
1200
1400
m/z
Cytokeratin 19
D
y8+1
R.DYSHYYTTIQDLR.D
100
y9+1
Relative Abundance
b11+1
b6+1
50
y12+2
b5+1
y2+1
y10+2
y11+2
y5+1
b9+1
y6+1
b4+1
b8+1
y10+1
y4+1
b10+1
b12+1
b7+1
y9+2
y11+1
y3+1
0
400
600
800
1000
1200
1400
m/z
Vimentin
C
y6+1
K.FADLSEAANR.N
100
Relative Abundance
y8+1
50
y5+1
y4+1
b5+1
b3+1
y3+1
b4+1
y7+1
y2+1
b9+1
b6+1
b2+1
y9+1
b8+2
b7+1
y1+1
b8+1
y9+2
0
200
300
400
500
600
700
800
900
m/z

## Slide 6
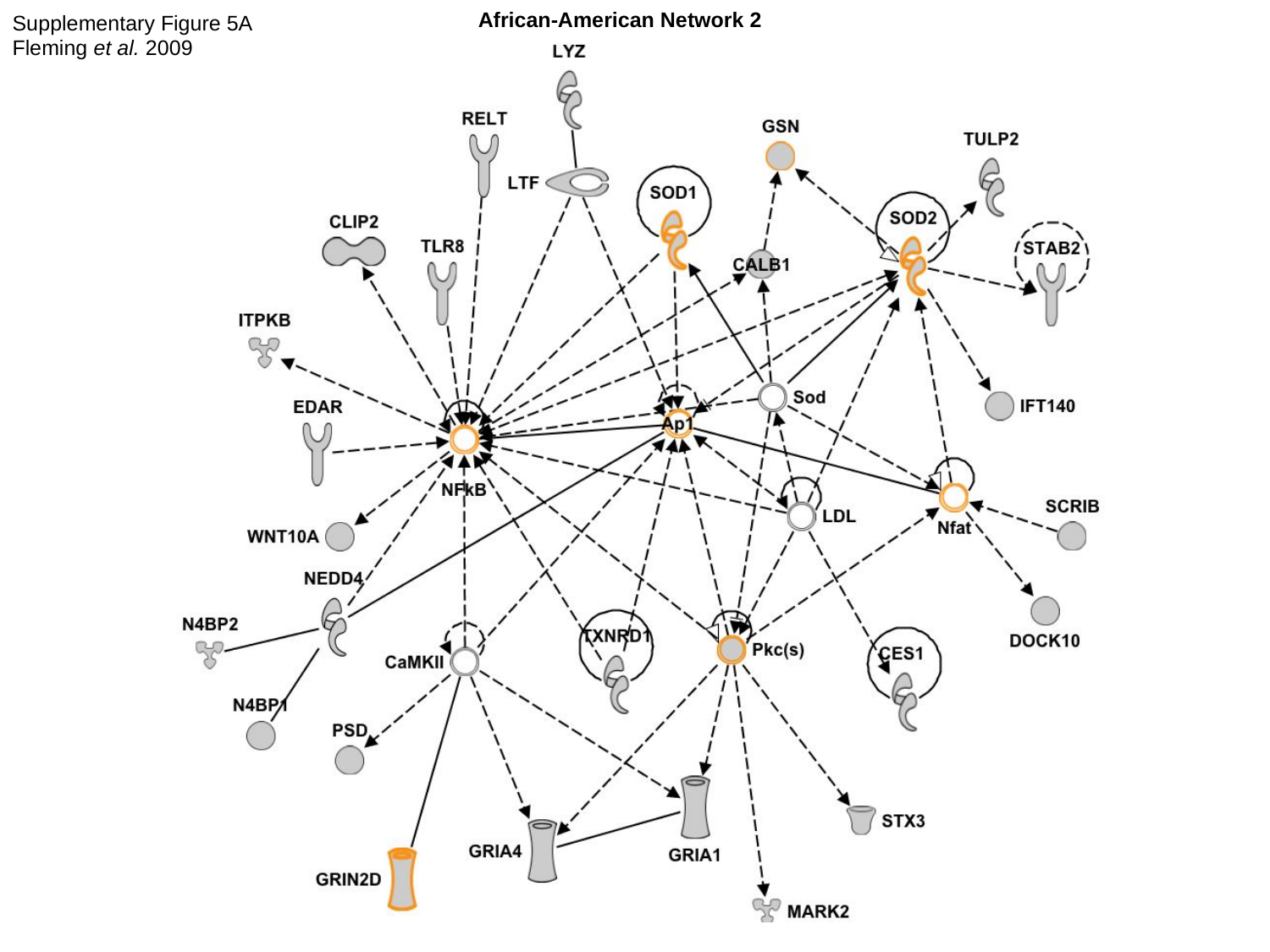

African-American Network 2
Supplementary Figure 5A
Fleming et al. 2009

## Slide 7
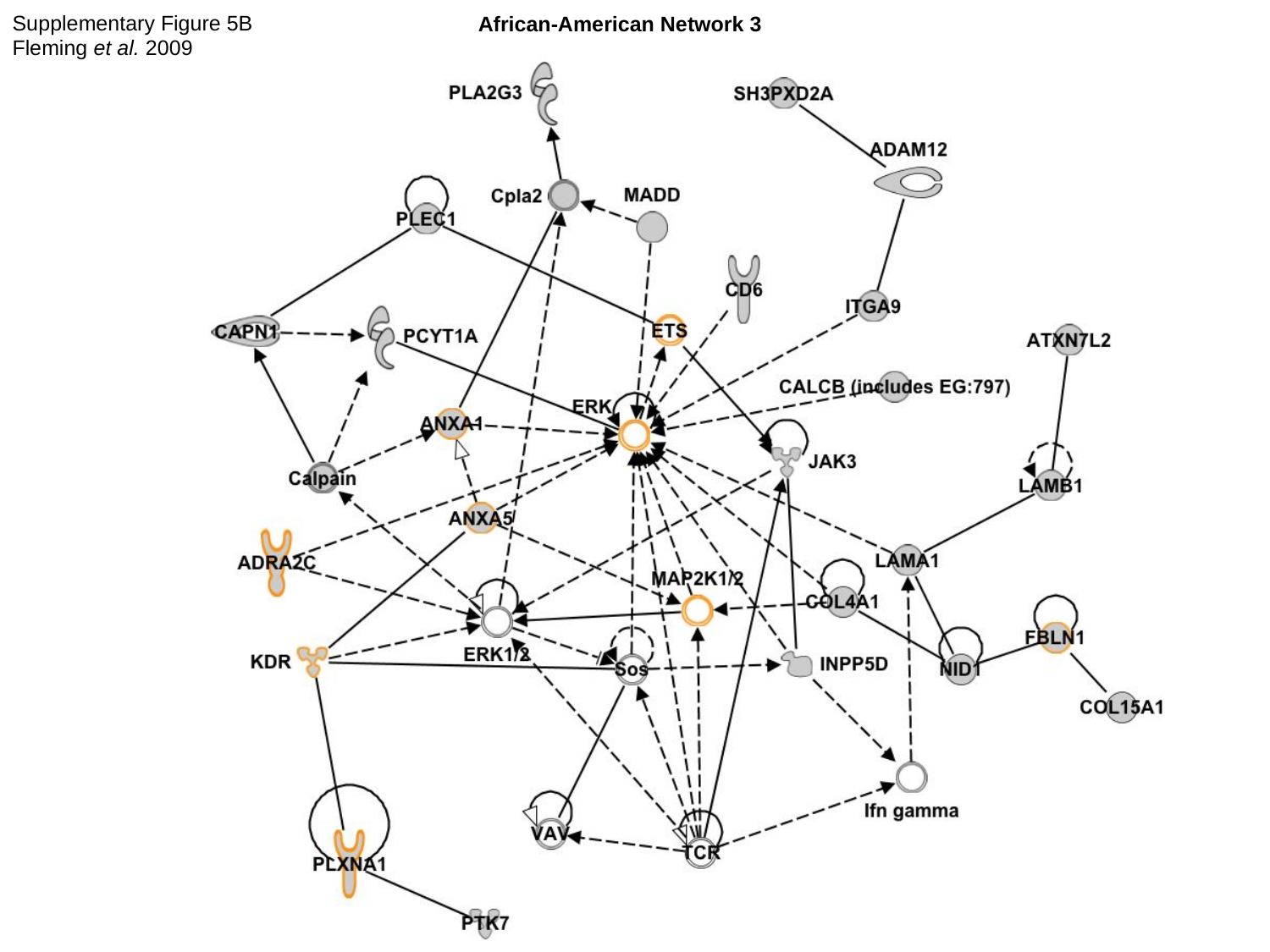

Supplementary Figure 5B
Fleming et al. 2009
African-American Network 3

## Slide 8
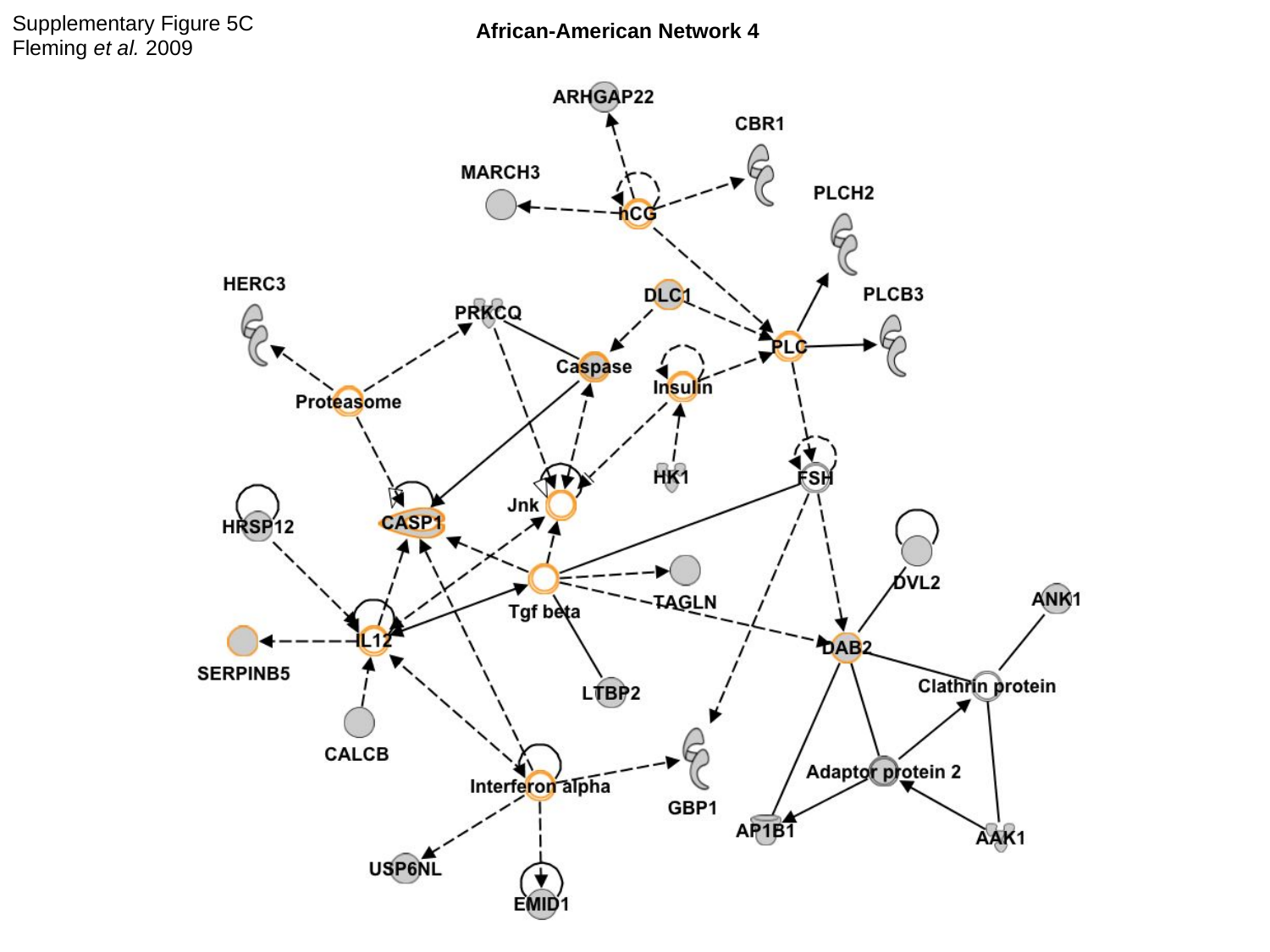

Supplementary Figure 5C
Fleming et al. 2009
African-American Network 4

## Slide 9
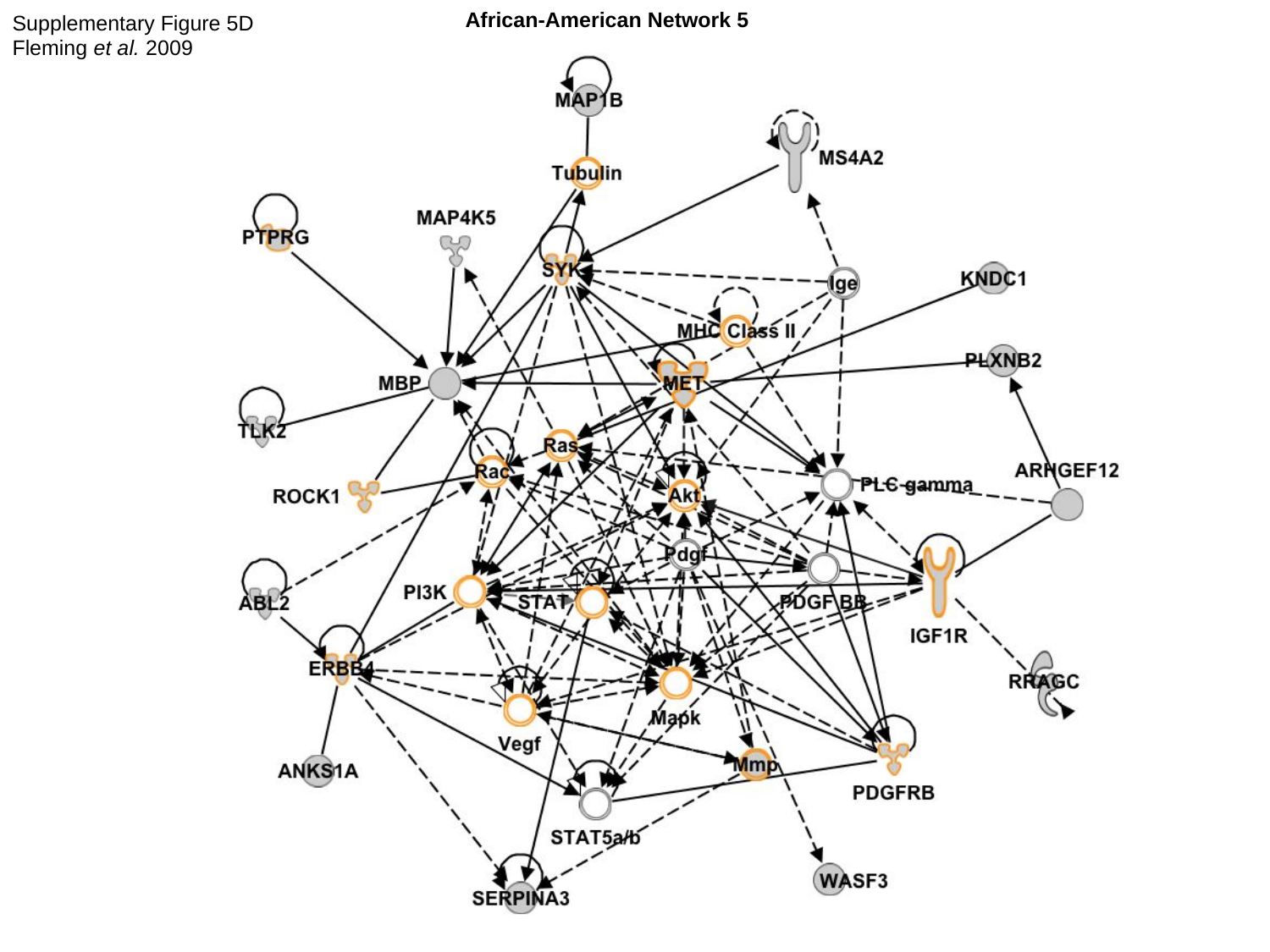

African-American Network 5
Supplementary Figure 5D
Fleming et al. 2009

## Slide 10
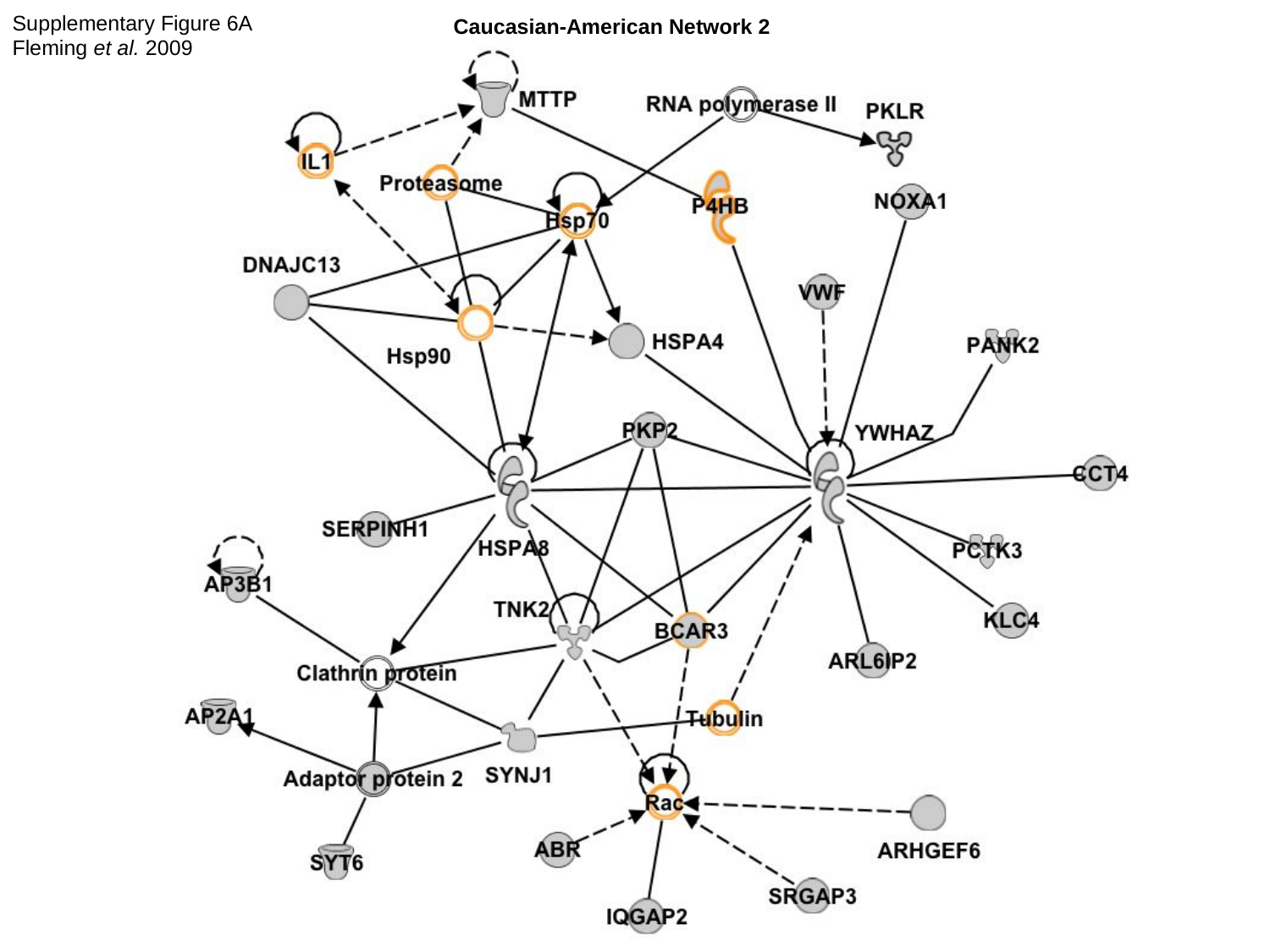

Supplementary Figure 6A
Fleming et al. 2009
Caucasian-American Network 2

## Slide 11
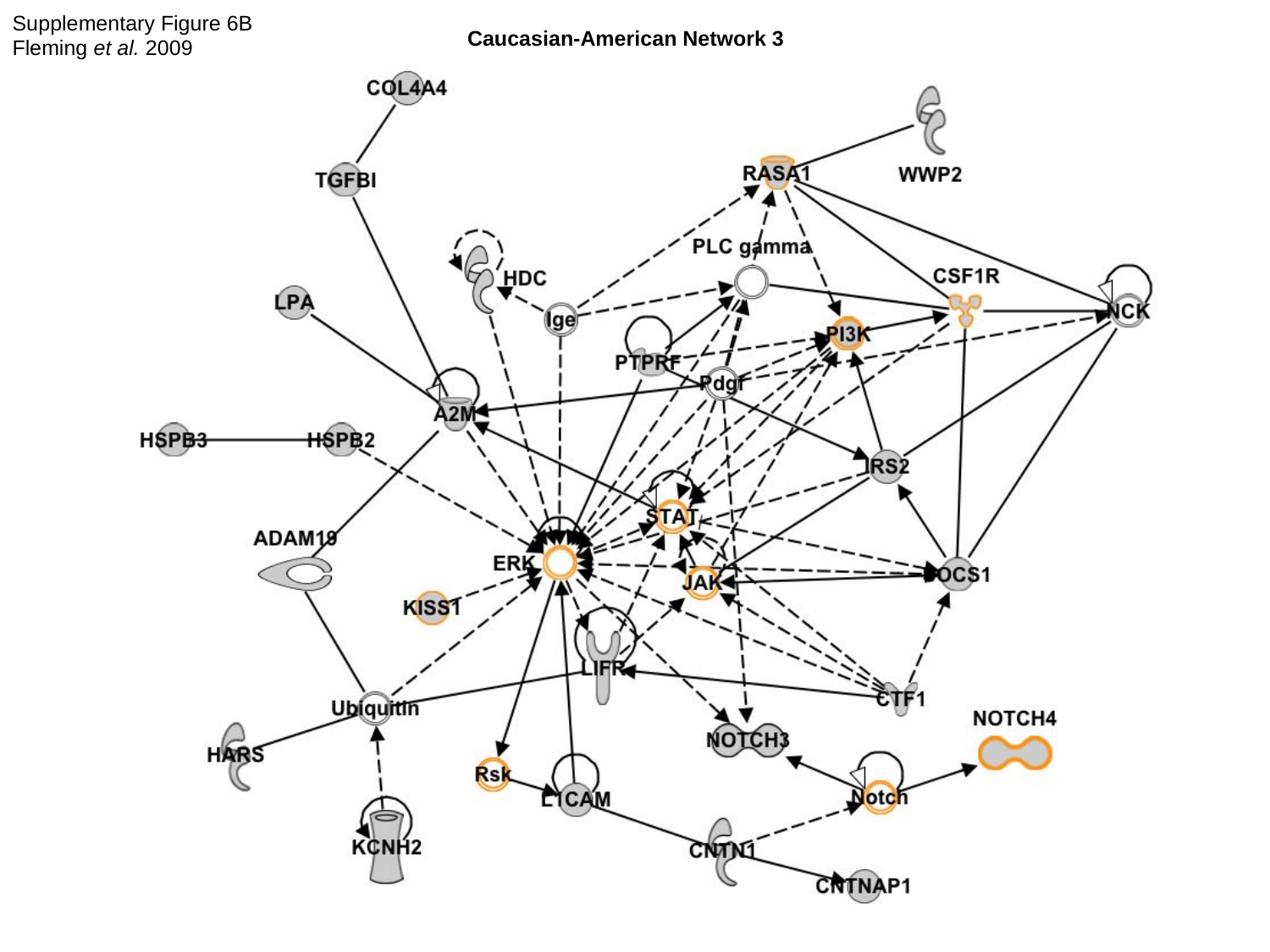

Supplementary Figure 6B
Fleming et al. 2009
Caucasian-American Network 3

## Slide 12
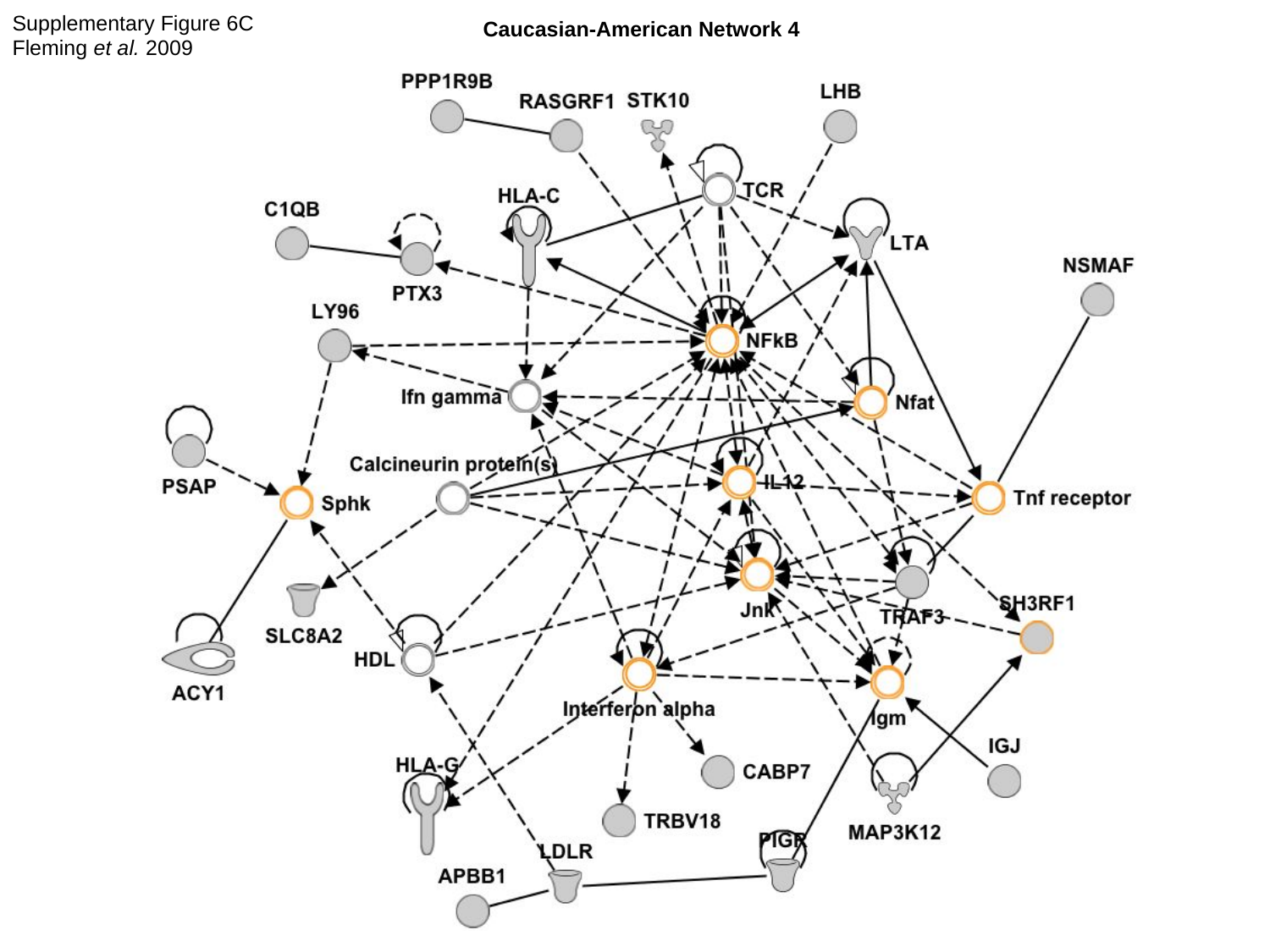

Supplementary Figure 6C
Fleming et al. 2009
Caucasian-American Network 4

## Slide 13
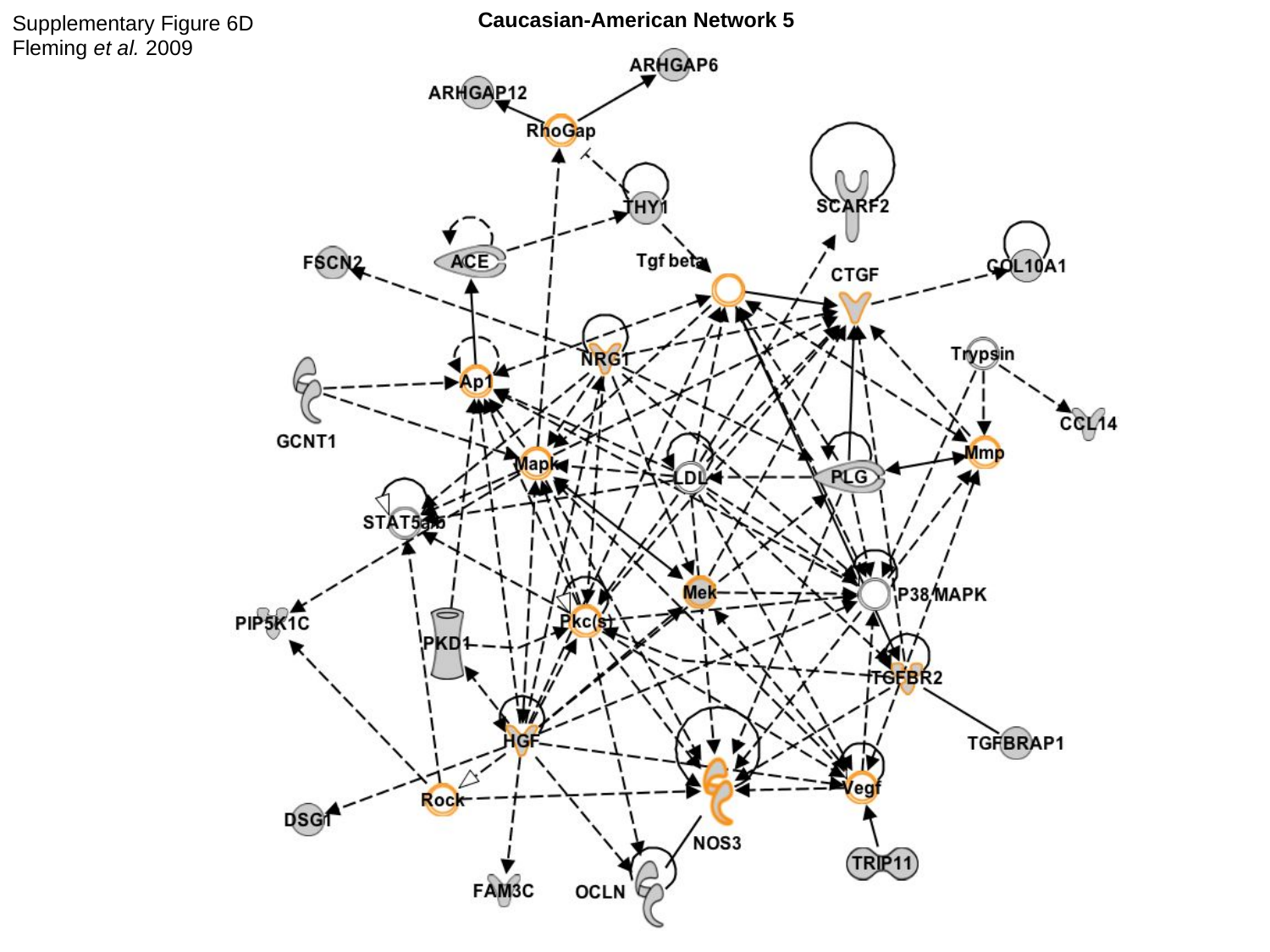

Caucasian-American Network 5
Supplementary Figure 6D
Fleming et al. 2009
